# Targeting Mitochondrial Network Disorganization is Protective in *C. elegans* Models of Huntington’s Disease

**DOI:** 10.14336/AD.2021.0404

**Published:** 2021-10-01

**Authors:** Emily Machiela, Paige D Rudich, Annika Traa, Ulrich Anglas, Sonja K Soo, Megan M Senchuk, Jeremy M Van Raamsdonk

**Affiliations:** ^1^Laboratory of Aging and Neurodegenerative Disease, Center for Neurodegenerative Science, Van Andel Research Institute, Grand Rapids MI 49503, USA; ^2^Department of Neurology and Neurosurgery, McGill University, Montreal, Quebec, H4A 3J1, Canada; ^3^Metabolic Disorders and Complications Program, and Brain Repair and Integrative Neuroscience Program, Research Institute of the McGill University Health Centre, Montreal, Quebec, H4A 3J1, Canada; ^4^Division of Experimental Medicine, Department of Medicine, McGill University, Montreal, Quebec, Canada; ^5^Department of Genetics, Harvard Medical School, Boston MA 02115, USA

**Keywords:** Huntington’s disease, mitochondria, mitochondrial dynamics, *C. elegans*, neuroprotection, genetics, neuroprotection, neurodegeneration, aggregation, DRP1, animal model

## Abstract

Huntington’s disease (HD) is an adult-onset neurodegenerative disease caused by a trinucleotide CAG repeat expansion in the *HTT* gene. While the pathogenesis of HD is incompletely understood, mitochondrial dysfunction is thought to be a key contributor. In this work, we used *C. elegans* models to elucidate the role of mitochondrial dynamics in HD. We found that expression of a disease-length polyglutamine tract in body wall muscle, either with or without exon 1 of huntingtin, results in mitochondrial fragmentation and mitochondrial network disorganization. While mitochondria in young HD worms form elongated tubular networks as in wild-type worms, mitochondrial fragmentation occurs with age as expanded polyglutamine protein forms aggregates. To correct the deficit in mitochondrial morphology, we reduced levels of DRP-1, the GTPase responsible for mitochondrial fission. Surprisingly, we found that disrupting *drp-1* can have detrimental effects, which are dependent on how much expression is decreased. To avoid potential negative side effects of disrupting *drp-1*, we examined whether decreasing mitochondrial fragmentation by targeting other genes could be beneficial. Through this approach, we identified multiple genetic targets that rescue movement deficits in worm models of HD. Three of these genetic targets, *pgp-3, F25B5.6* and *alh-12*, increased movement in the HD worm model and restored mitochondrial morphology to wild-type morphology. This work demonstrates that disrupting the mitochondrial fission gene *drp-1* can be detrimental in animal models of HD, but that decreasing mitochondrial fragmentation by targeting other genes can be protective. Overall, this study identifies novel therapeutic targets for HD aimed at improving mitochondrial health.

Huntington’s disease (HD) is an autosomal dominant neurodegenerative disease caused by an expansion of the polyglutamine tract in the N-terminal of the huntingtin (Htt) protein. The expression of the expanded polyglutamine tract is both necessary and sufficient for cellular toxicity [1], although loss of wild-type huntingtin function might also contribute to disease pathogenesis [2]. HD is characterized by progressive cognitive decline, neuropsychiatric abnormalities, and motor impairment [3]. In unaffected individuals, the polyglutamine tract of the Htt protein is polymorphic, containing from 9-34 glutamines. However, mutations in the HD gene leading to 35 or more glutamines have been shown to cause HD. Within the disease range of 35 glutamines and above, age of onset negatively correlates with the number of glutamines present [4, 5]. Although Htt is expressed in every cell of the body and pathology has been observed in multiple tissues, cellular dysfunction and atrophy are most severe in the GABAergic medium spiny neurons of the striatum. The reasons for this selective vulnerability are still unknown.

While the cause of cellular dysfunction in HD is still incompletely understood, mitochondrial dysfunction is thought to play a central role in disease pathogenesis [6, 7]. There is significant evidence for mitochondrial dysfunction in HD patients and animal models including decreased activity of complexes in the electron transport chain [8], increased lactate production in the brain [9], decreased levels of ATP production [10], decreased mitochondrial membrane potential [11], and impaired trafficking of mitochondria within the cell [12]. The importance of mitochondrial dysfunction to HD pathogenesis is also suggested by the fact that systemic administration of 3-nitropropionic acid, a neurotoxin that inhibits mitochondrial function, can reproduce symptoms and neuropathological deficits that occur in HD [13, 14]. In addition, a genome-wide association study investigating genetic modifiers for age of onset of HD found pathways involving mitochondrial fission to significantly modify age of onset of the disease [15].

Recent work has demonstrated that mitochondrial dynamics are disrupted in HD. Mitochondria continually change their shape in response to the needs of the cell, and these changes impact both the function and distribution of the mitochondria. Mitochondrial morphology is determined by two opposing processes: fission and fusion. Mitochondrial fission results in an increase in mitochondrial fragmentation as new mitochondria are pinched off of existing mitochondria or mitochondrial networks. The fission process is mediated by dynamin-related protein 1 (DRP-1/DRP1) with the help of mitochondrial fission proteins (FIS-1/FIS-2/FIS1) and mitochondrial fission factors (MFF-1/MFF-2/MFF1). Conversely, mitochondrial fusion leads to decreased mitochondrial fragmentation by joining individual mitochondria together with other mitochondria to form interconnected mitochondrial networks. The fusion process requires merging of the inner mitochondrial membrane by optic atrophy protein 1 (EAT-3/OPA1) and the merging of the outer mitochondrial membrane by mitofusin (FZO-1/MFN).

In HD cell lines [16-21], the 3-nitropropionic acid neurotoxin model of HD [22], cells from HD mouse models [23] and cells derived from HD patients [19, 23], it has been found that mitochondria are more fragmented than in unaffected controls. In addition, examination of mitochondria by electron microscopy in brain sections from R6/2 mice [20] and YAC128 mice [17] revealed the presence of smaller mitochondria in HD mice compared to controls, suggesting that increased mitochondrial fragmentation also occurs *in vivo*. In these studies, it has been shown that the expression of exon 1 fragments of mutant Htt is sufficient to cause mitochondrial fragmentation [16-18]. The increase in mitochondrial fragmentation in HD could result from excess mitochondrial fission, decreased mitochondrial fusion or both. While the precise mechanism by which mutant Htt causes mitochondrial fragmentation is still unclear, contributing factors may include: alterations in expression levels of fission and fusion proteins [20, 24, 25], an increase in DRP-1 enzymatic activity resulting from increased interaction with mutant Htt [17, 26], increased S-nitrosylation of DRP-1 leading to increased fission activity [18], increased levels of reactive oxygen species [22, 27], decreased Nrf2 signaling [20], and increased calcineurin activity [23].

Importantly, reducing mitochondrial fragmentation has been shown to be beneficial in models of HD. Decreasing the activity or expression of the mitochondrial fission protein DRP-1 increases survival in cell models of HD [16, 17, 19]. In addition, treating a worm model of HD expressing exon 1 fragment of mutant Htt with 74 CAG repeats in body wall muscle with RNAi against *drp-1* improved the movement deficit present in these worms, although the effect of this treatment on mitochondrial morphology in these worms was not assessed [16]. Furthermore, treatment of the R6/2 mouse model of HD with a DRP-1 inhibitor (P110-Tat) improved behavior, survival and neuropathology in these mice, and resulted in a significant increase in cristae area in electron micrographs [19]. The P110-Tat DRP-1 inhibitor was also able to ameliorate mitochondrial structural deficits in the hearts of R6/2 mice, indicating that this inhibitor can also be effective in muscle tissue [21]. Combined, these results suggest that developing interventions that inhibit DRP-1 may be beneficial in the treatment of HD.

In this work, we explore the role of mitochondrial fragmentation in the pathogenesis of HD, and whether targeting this deficit may be an effective strategy to treat HD. To do this, we use *C. elegans* models, which permit the visualization of mitochondrial morphology in a live organism that exhibits quantifiable, disease-relevant phenotypic deficits. We find that *C. elegans* models of HD exhibit mitochondrial fragmentation, which is temporally correlated with polyglutamine aggregation. We find that decreasing levels of *drp-1* fails to correct the deficit in mitochondrial morphology and can be detrimental, depending on the level of disruption. In contrast, treating worms with other RNAi clones that decrease mitochondrial fragmentation improved movement in worm models of HD.

## MATERIALS AND METHODS

### Strains

The following strains were used in this study:

N2 (WT)

JVR240 *syIs243[Pmyo-3::TOM20:RFP]* referred to as mitoRFP

MQ1699 *Punc-54::Htt28Q:GFP* referred to as BW-Htt28Q

MQ1698 *Punc-54::Htt74Q:GFP* referred to as BW-Htt74Q

AM138 *rmIs120[Punc-54::24Q:YFP]* referred to as BW-24Q

AM141 *rmIs133[Punc-54::40Q:YFP]* referred to as BW-40Q

MQ1753 *drp-1* (*tm1108*)

JVR248 *Punc-54::Htt28Q:GFP;syIs243[Pmyo-3::TOM20:RFP]*

JVR250 *Punc-54::Htt74Q:GFP;syIs243[Pmyo-3::TOM20:RFP]*

JVR251 *drp-1*(*tm1108*);*Punc-54::Htt74Q:GFP*

JVR255 *drp-1*(*tm1108*);*syIs243[Pmyo-3::TOM20:RFP]*

JVR259 *drp-1(tm1108);Punc-54::Htt74Q:GFP;syIs243[Pmyo-3::TOM20:RFP]*

JVR473 *syIs243[Pmyo-3::TOM20:RFP];rol-6(su1006)*

JVR474 *rmIs120[Punc-54::24Q:YFP];syIs243[Pmyo-3::TOM20:RFP];rol-6(su1006)*

JVR475 *rmIs133[Punc-54::40Q:YFP];syIs243[Pmyo-3::TOM20:RFP];rol-6(su1006)*

JVR463 *Punc-54::Htt74Q:GFP;syIs243[Pmyo-3::TOM20:RFP];rol-6(su1006)*

JVR520 *Punc-54::Htt28Q:GFP;syIs243[Pmyo-3::TOM20:RFP]; rol-6(su1006)*

JVR521 *drp-1(tm1108);Punc-54::Htt74Q:GFP;syIs243[Pmyo-3::TOM20:RFP]; rol-6(su1006)*

All strains were maintained at 20°C on NGM plates seeded with OP50 bacteria. All crosses were confirmed by genotyping using PCR and, where applicable, confirmed by fluorescent microscopy.

### Confocal imaging and quantification

Mitochondrial morphology was imaged and quantified using worms that express mitochondrially-targeted RFP specifically in body wall muscle (*syIs243[Pmyo-3::TOM20:RFP]*). In order to facilitate imaging, these worms were crossed into a *rol-6* background. The *rol-6* mutation results in animals moving in a twisting motion, thus displaying a helix of muscle cells upon imaging (Supplementary Fig. 1). This is beneficial when imaging the mitochondria of body wall muscle cells in the nematode as it ensures that several portions of both the ventral and dorsal quadrants of muscle cells are within the plane of view (facing the objective lens). This facilitates mitochondrial imaging as the tubular mitochondrial organization can be seen within much of the muscle. Without the *rol-6* mutation, the lateral side of the nematode may face the objective lens with only the longitudinal edges of the muscle being visible, thus making it difficult to observe mitochondrial organization.

To image the mitochondria, approximately 20 young adult worms were mounted on 2% agar pads and immobilized using 10 µM levamisole. Worms were imaged under a 63x objective lens on a Zeiss LSM 780 or Nikon A1R Ti confocal microscope. All conditions were kept the same for all images. For representative images, a z-stack of images spaced 0.125-0.40 µm apart were collected and a z-stack projection was created using either Nikon Elements or ImageJ to compress stacks into a single image.

For quantification, a single plane image taken in the same body region for each worm was used to avoid the complication of mitochondria being present in two planes. This slice was made binary using the Nikon Elements thresholding tool. A background subtraction of a constant 50 was applied. Next, a pixel picker was applied to several control mitoRFP images to define the low and high threshold levels. Once optimum threshold numbers were defined for control images, these limits were applied to all images to be quantified with the separate function on. Size and circularity were not used to define thresholds. Prior to creating the binary mask, images were manually inspected for proper threshold parameters. In the event that threshold parameters mislabeled mitochondria, objects were manually included or excluded prior to masking. Mitochondrial circularity, number, and area were measured using the measure objects tool in Nikon Elements AR after the threshold mask was applied. For mitochondrial circularity, raw numbers were exported to Microsoft Excel and averages were calculated prior to statistical analysis in Graphpad Prism. All other calculations were exported directly to Prism for analysis.

### Oxygen consumption

Basal oxygen consumption rate was measured using a Seahorse XF_e_96 analyzer (Seahorse bioscience Inc., North Billerica, MA, USA)[28]. Synchronized worms at day 1 of adulthood were cleaned in M9 buffer (22?mM KH_2_PO_4_, 34?mM NA_2_HPO_4_, 86?mM NaCl, 1?mM MgSO_4_). Cleaned nematodes were pipetted in calibrant (~50 worms per well) into a Seahorse 96-well plate. Oxygen consumption was measured six times and rates of respiration were normalized to the number of worms in each individual well. The plate readings were begun within 20 minutes of introduction of the worms into the well. Reading from each well were normalized relative to the number of animals per well. Well probes were hydrated in a 175 µL Seahorse calibrant overnight before this assay was begun. We found it is important to turn off the heating incubator to allow the Seahorse machine to reach room temperature before placing nematodes inside the machine. For these experiments, we chose to measure oxygen consumption per worm so that we could compare the rate of oxidative phosphorylation for the whole organism to the whole organism phenotypes that we were measuring (e.g. movement and lifespan). Also, we chose to use a Seahorse extracellular flux analyzer to measure oxygen consumption so that all of the strains being compared could be measured at the same time so that the conditions would be identical. With the low number of worms that are used in each well, it would be difficult to accurately measure protein content, especially given that worms will often stick to pipet tips or the side of the dish during transfer.

### ATP production

ATP levels were measured using a luminescence-based ATP kit [29]. Approximately 200 worms were age-synchronized by a limited lay. Worms were collected in de-ionized water, washed, and freeze-thawed three times. The resulting pellet was sonicated in a Bioruptor (Diagenode) with 30 cycles of 30 seconds on, 30 seconds off. The pellet was boiled for 15 minutes to release ATP, then spun at 4°C at 11,000 × *g* for 10 minutes. The supernatant was collected and measured using a Molecular Probes ATP determination Kit (Life Technologies). Luminescence was normalized to protein content, which was measured with a Pierce BCA protein determination kit (Thermo Scientific).

### Rate of movement

For measuring the effects of drp-1, the rate of movement was assessed by measuring thrashing rate in liquid using video-tracking and computer analysis [30]. Approximately 50 pre-fertile day 1 young adult worms were placed in M9 buffer on a clean NGM plate. Videos were taken with an Allied Vision Tech Stingray F-145 B Firewire Camera (Allied Vision, Exton, PA, USA) at 1024×768 resolution, 8-bit using the MATLAB image acquisition toolbox. Analysis was performed using wrMTrck plugin for ImageJ (publicly available at www.phage.dk/plugins).

For screening the mitochondrial fragmentation genes, the rate of movement was assessed by measuring absolute crawling speed and thrashing rate using WormLab 2019.1.2 (MBF BioSciences). Experiments were done on day 1 adults, and the animals were isolated as L4’s 24 hrs before. Animals were exposed to RNAi using the parental paradigm, or if the RNAi clone inhibited development, the animals were grown on empty vector RNAi and placed on the respective RNAi clone as L4’s (E04A4.4, C33A12.1, abhd-11.1, iars-1, his-12 and acs-1). For the experiment, worms were removed from their plates with M9 buffer, washed twice with M9 buffer, and placed on clean 3-cm NGM plates. A Kimwipe was used to remove excess liquid and the worms were allowed to acclimate for 5 minutes. The plates were placed under a monochrome digital camera (Basler acA2440 camera with an AF Micro Nikkor 60 mm f/2.8 D lens) and tapped to stimulate movement. 20-30 animals were normally in frame. The worms were recorded using the WormLab software (Version 2019.1.2), in 45 s long videos with a resolution of 2456x2052 at a scale of 9.9 μm/pixel. Crawling was recorded at a frame rate of 7.5 frames/s. After recording crawling, M9 buffer was added to the plate. Worms were allowed to acclimate for 5 minutes and then recorded while swimming at a frame rate of 14 frames/s. Worms that were tracked for less than half of the video were excluded from the analysis because the worms could have left the field-of-view and then returned, causing double counting. Crawling speed and thrashing rates were analyzed using the Absolute Peristaltic Speed results and Wave Initiation Rate results, respectfully, exported from WormLab and processed using Microsoft Excel 2016 (Microsoft, Redmond, WA, USA). 3 replicates of ~20 worms were performed for each RNAi clone.

### Imaging and quantification of aggregation

Experimental animals were day 1 adults and were isolated at the L4 stage 24 hours before the experiments. Animals were exposed to RNAi using the parental paradigm, or if the RNAi clone inhibited development, the animals were grown on empty vector RNAi and placed on the respective RNAi clone as L4’s (*E04A4.4*, *C33A12.1*, *abhd-11.1*, *iars-1*, *his-12* and *acs-1*). 10 animals were mounted on 3% agarose pads using 10mM levamisole for anesthesia and imaged within 45 minutes of levamisole exposure. Images were taken on a Nikon Eclipse Ti microscope with a Nikon Plan Apo 20x/0.75 NA objective and a Zyla Andor sCM05 camera. z-stacks of the animals were recorded with a 2μm step size. Analysis was performed in ImageJ (Version 2.1.0/1.53c) by merging the z-stack using Temporal-Color Code to color code the different z planes, and then the aggregates were manually counted using the Cell Counter plugin. At least 10 animals per clone were quantified. Clones which caused a measurable decrease were repeated for confirmation.

### Lifespan

Lifespan was determined on nematode growth media (NGM) agar plates with 25?μM 5-fluoro-2′-deoxyuridine (FUdR) in order to reduce the development of progeny. Plates with 25 μM FUdR do not completely prevent the development of progeny to adulthood in the first generation so animals were transferred to fresh agar plates after 4 days [31]. After the initial transfer, worms were moved to fresh plates weekly. Animal survival was observed every 2 days by gentle prodding. Three replicates of 30 animals each were completed.

### Brood size

Brood size was determined by placing individual young adult staged animals onto agar plates with daily transfers to new plates until progeny production ceased. The resulting progeny were allowed to develop to adulthood before quantification. Three replicates of 5 animals each were completed.

### Post-embryonic development

Post-embryonic development (PED) was assessed by moving eggs to agar plates. After 3?hours, newly hatched L1 worms were transferred to a new plate. The hours from hatching to the young adult transition was measured as the PED time. Three replicates of 20 animals each were completed.

### Quantitative reverse-transcription PCR (qPCR)

mRNA was collected from pre-fertile young adult worms using Trizol as previously described [32]. We collected three biological replicates each for WT, BW-40Q and BW-Htt74Q worms. The mRNA was converted to cDNA using a High-Capacity cDNA Reverse Transcription kit (Life Technologies/Invitrogen) according to the manufacturer’s directions. qPCR was performed using a FastStart Universal SYBR Green kit (Roche) in an AP Biosystems real-time PCR machine [33, 34]. Primer sequences utilized:

*drp-1* L-GGTTTTCACAGACTTCGATGC

R-TA GGCTCCGAAGTAGCGAAA

*fis-1* L-AGAAATTCTGGCGGCTCGT

R-GCG TGTGCAAGAGCAAGATA

*fis-2* L-GGGAATCGTGTGTCTTGAGAAG

R-GG CCATCATGAGTCATTGC

*mff-1* L-CCGCTCAATAGATTGATGAACA

R-T TGGGGACTTCCATTCTGAG

*mff-2* L-TGGATAAACTTCCAACGGAAA

R-CC GGGCTGTGTCTAGCTCT

*eat-3* L-GCGGCTAGAACGTGGTATGA

R-CGG GCTCTTTTACTGGAACA

*fzo-1* L-GCTTTCTGCAGGTTGAAGGT

R-CGA CACCAGGGCTATCAAGT

*gfp* L-GACGACGGCAACTACAAGAC

R-TCC TTGAAGTCGATGCCCTT

### Quantification of mitochondrial DNA

Mitochondrial:nuclear DNA ratios were calculated as previously described with minor modifications [35]. Wild-type, BW-40Q and BW-Htt74Q worms were grown on OP50 at 20°C. Animals were isolated at the L4 developmental stage and collected the following day as day 1 adults, where three replicates of six worms for each strain were isolated in 90 µl of worm lysis buffer and lysed. The lysed worm samples were analyzed by quantitative RT-PCR as previously described for *C. elegans* [35], using established mitochondrial-DNA specific *nd-1* primers and nuclear-DNA specific *cox-4* primers [35, 36]. Samples were collected three times, each time with three biological replicates. We performed three technical replicates on each of these samples. The primer sequences used are as follows:

*nd-1* L-AGCGTCATTTATTGGGAAGAAGAC

R-AAGCTTGTGCTAATCCCATAAATGT

*cox-4* L-GCCGACTGGAAGAACTTGTC

R-GCG GAGATCACCTTCCAGTA

### RNA interference (RNAi)

All RNAi clones were sequence verified. To knockdown expression of genes, the RNAi clones were grown approximately 12 hours in LB with 50 μg/ml carbenicillin. Cultures were concentrated (5x) and seeded onto NGM plates containing 5 mM IPTG and 50 μg/ml carbenicillin. Plates were incubated to induce RNAi for 2 days at room temperature. RNAi was performed at 20°C. For experiments examining RNAi knockdown beginning in the parental generation (L4 parental paradigm), L4 worms were plated on RNAi plates, transferred to a new plate the following day as gravid adults, and then removed after 24 hours. The progeny from these worms were used for analysis.

### Quantification of knockdown of drp-1 mRNA by drp-1 RNAi

Wild-type and BW-Htt74Q worms were grown on empty vector (EV) and *drp-1* RNAi following the RNAi parental paradigm described in the RNAi methods. Levels of *drp-1* and *act-3* were quantified through quantitative RT-PCR. Primers were specifically designed to exclude the *drp-1* RNA used to induce RNAi (*drp-*1: L-GAGATGTC GCTATTATCGAACG, R-CTTTCGGCACACTATCC TG). Three biological replicates each with three technical replicates were performed.

### Effect of RNAi clones on mitochondrial morphology

In order to examine the effect of RNAi clones on mitochondrial morphology in *mitoRFP;rol-6* and *BW-Htt74Q;mitoRFP;rol-6* worms, 30 L4 worms were picked to plates seeded with EV, *pgp-3, F25B5.6,* and *alh-12* RNAi-expressing bacteria. After 24 hours gravid adults were picked to new RNAi plates. After another 24 hours gravid adults were removed from the RNAi plates. Progeny were allowed to develop to the young adult stage before imaging.

### Experimental design and statistical analysis

Experiments were performed such that the experimenter was blinded to the genotype of the worms. Experimental worms were randomly selected from maintenance plates. For all experiments, we completed a minimum of three biological replicates (independent population of worms tested on a different day). Where possible (*e.g*., measurement of movement) assays were completed using automated approaches with computer analysis to eliminate any potential experimental bias. We did not perform power calculations to determine the N required for experiments as the Ns that are used in *C. elegans* experiments are typically much greater than required to identify a statistically significant difference. For measurements of mitochondrial morphology, we used 8 biological replicates. For oxygen consumption we performed at least 8 replicates with ~50 worms per replicate. For ATP measurements, we performed 3 biological replicates with ~200 worms per replicate. For mRNA measurements, we used 3 biological replicates of a full 60 mm plate of worms. For the thrashing assays, we quantified movement in at least 40 worms over 3 biological replicates. For lifespan assays, we completed three biological replicates with at least 30 worms per replicate. Brood size was measured in 6 worms individually. Post-embryonic development time was measured in 3 replicates of 25 worms per replicate. Statistical significance of differences between groups was determined by one-way, two-way or repeated measures ANOVA using Graphpad Prism. Lifespan data were graphed using a Kaplan-Meier survival plot and the significance of differences between two plots was determined using the Log-rank test. Error bars indicate standard error of the mean. This study was not pre-registered. No sample size calculations were performed. This study did not include a pre-specified primary endpoint.

## RESULTS

### Mitochondrial networks are disrupted in C. elegans models of Huntington’s disease

In order to study the relationship between mitochondrial fragmentation and disease pathogenesis, we first sought to determine if mitochondrial dynamics are disrupted in worm models of HD. To visualize the morphology of the mitochondria, we crossed two different worm models of HD to mitoRFP worms, which express the mitochondrial targeting sequence of TOM-20 linked to RFP under the body wall muscle *myo-3* promoter (*Pmyo-3::TOM-20:RFP*). The first is a worm model of HD that expresses an exon 1 fragment of human huntingtin (Htt) connected to either an unaffected-length 28Q or disease-length 74Q repeats tagged with GFP in body wall muscle, which will be referred to as BW-Htt28Q and BW-Htt74Q, respectively. Both of these lines have been characterized previously [16, 37].

**Figure 1.**
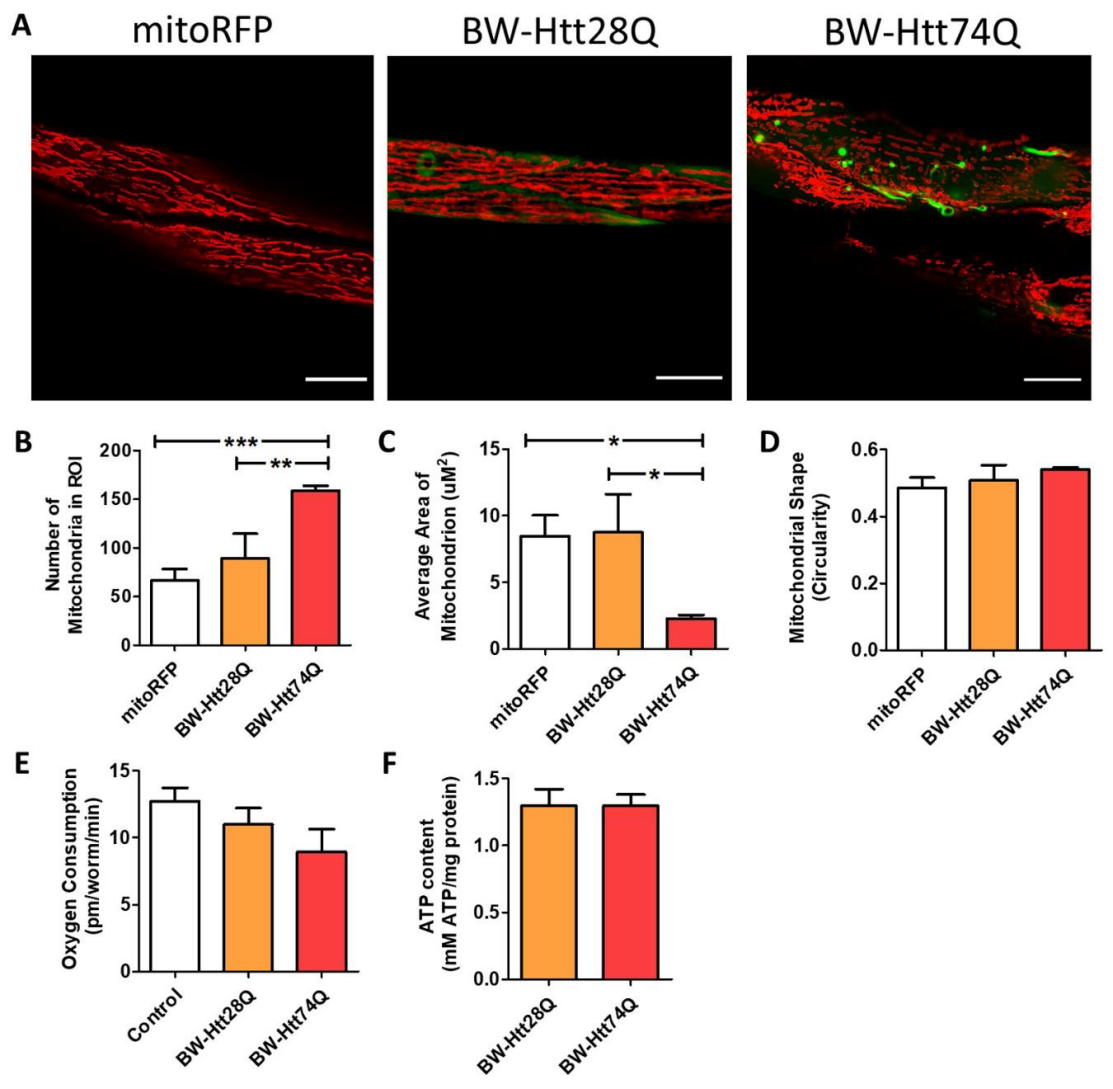
Mitochondrial networks are disrupted in *C. elegans* models of Huntington’s disease. Worms expressing an expanded, disease-length polyglutamine tract of 74Q in body wall muscle (BW-Htt74Q worms) exhibit mitochondrial fragmentation and mitochondrial network disorganization (see Supplementary Fig. 2). In contrast, worms expressing a shorter, unaffected-length polyglutamine tract of 28Q (BW-Htt28Q worms) have tubular mitochondria, similar to control worms (mitoRFP worms) (A). Mitochondria are labelled with RFP (red), while Htt is labelled with GFP (green). mitoRFP strain is *syIs243[Pmyo-3::TOM20:RFP].* BW-Htt28Q and BW-Htt74Q worms also express *syIs243[Pmyo-3::TOM20:RFP]* transgene. The images shown are from a single focal plane collected on a confocal microscope. Scale bars indicate 15 µM. Quantification of mitochondrial morphology at day 1 of adulthood reveals that BW-Htt74Q worms have an increased number of mitochondria (B) and decreased average mitochondrial area (C), both of which are consistent with increased mitochondrial fragmentation. Mitochondrial shape is not significantly changed in BW-Htt74Q worms compared to BW-Htt28Q and mitoRFP control worms (D). Despite the disruption of mitochondrial morphology, whole worm oxygen consumption (E) and ATP levels (F) are unchanged in BW-Htt74Q worms. A minimum of three biological replicates were performed. Bars indicate the mean value. One-way ANOVA was used to assess significance. Error bars indicate SEM. ROI - region of interest. *p<0.05, **p<0.01, ***p<0.001.

To image these worms, we used confocal microscopy in live, immobilized worms, as we and others have done previously [28, 38-40]. While wild-type worms exhibit parallel tracts of elongated mitochondria in their body wall muscle cells, BW-Htt74Q worms, which express a disease-length polyglutamine tract, exhibit mitochondrial fragmentation (Fig. 1A) and disorganized mitochondrial networks (Supplementary Fig. 2) at day 1 of adulthood. In contrast, there was no change in mitochondrial structure in BW-Htt28Q worms, which express an unaffected-length polyglutamine tract (Fig. 1A; Supplementary Fig. 2). Quantification of mitochondrial morphology revealed that BW-Htt74Q worms have a significantly increased number of mitochondria (Fig. 1B) and decreased mitochondrial area (Fig. 1C) compared to wild-type mitochondria, but mitochondrial shape was unaffected (Fig. 1D).

An increase in mitochondrial number could result from mitochondrial biogenesis or mitochondrial fragmentation. To distinguish between these two possibilities, we measured mitochondrial DNA (mtDNA) content. We found that mtDNA content in BW-Htt74Q worms is the same as wild-type worms (Supplementary Fig. 3). This suggests that the increase in mitochondrial number results from fragmentation of existing mitochondria, not increased mitochondrial biogenesis.

To determine how the disruption of mitochondrial networks in BW-Htt74Q worms affects the function of the mitochondria, we measured basal oxygen consumption and ATP levels at day 1 of adulthood. Although mitochondrial morphology is clearly disrupted in BW-Htt74Q worms, both oxygen consumption (Fig. 1E) and ATP levels (Fig. 1F) were equivalent to wild-type levels in these worms. However, this result should be interpreted cautiously as these are whole worm measurements in a model in which the mutant Htt exon 1 fragment protein is only expressed in the 95 body wall muscle cells out of 959 total cells in the worm.

**Figure 2.**
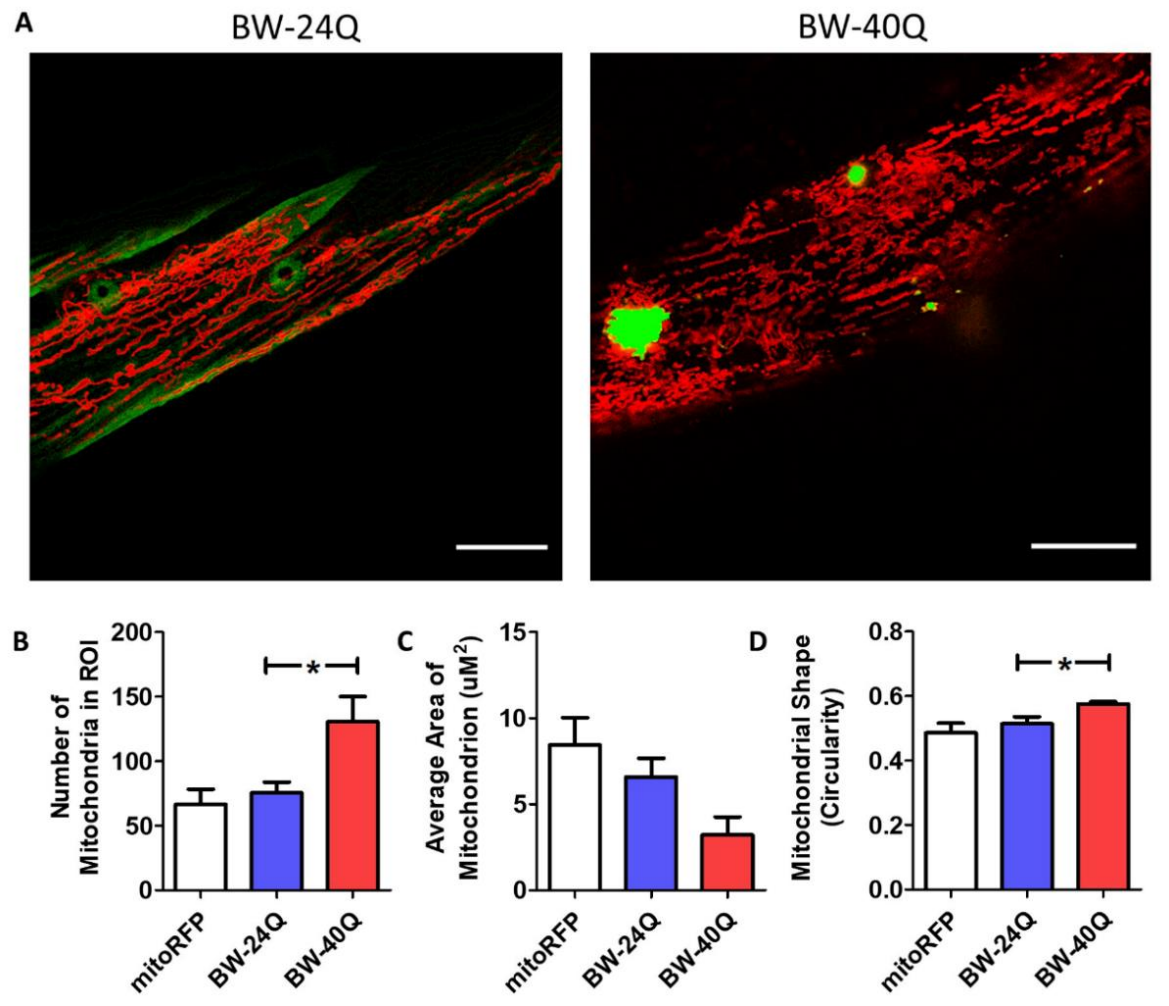
Mitochondrial networks are disrupted in BW-40Q worm model of Huntington’s disease. Worms expressing an expanded, disease-length polyglutamine tract of 40Q in body wall muscle (BW-40Q worms) exhibit mitochondrial fragmentation and disorganized mitochondrial networks (see Supplementary Fig. 4). In contrast, worms expressing a shorter, unaffected-length polyglutamine tract of 24Q (BW-24Q worms) have tubular mitochondria, similar to control worms (mitoRFP worms) (A). Mitochondria are labelled with RFP (red), while polyglutamine protein is labelled with YFP (green/yellow). BW-24Q and BW-40Q worms express *syIs243[Pmyo-3::TOM20:RFP]* transgene. The images shown are from a single focal plane collected on a confocal microscope. Scale bars indicate 15 µM. Quantification of mitochondrial morphology at day 1 of adulthood reveals that BW-40Q worms have an increased number of mitochondria (B) and a trend towards decreased average mitochondrial area (C). Mitochondrial circularity is significantly increased in BW-40Q worms compared with wild-type worms (D). A minimum of three biological replicates were performed. Bars indicate the mean value. One-way ANOVA was used to assess significance. Error bars indicate SEM. ROI - region of interest. *p<0.05.

To determine the extent to which the disruption of the mitochondrial network is dependent on the presence of Htt exon 1 fragment, we also examined mitochondrial morphology in a model of HD that expresses a pure polyglutamine tract. We examined worms that express either an unaffected (24 glutamines) or disease-length (40 glutamines) polyglutamine tract tagged with YFP in body wall muscle under the *unc-54* promoter. These worms will be referred to as BW-Q24 and BW-Q40 worms, respectively. Both lines are integrated and previously characterized [41].

As with BW-Htt74Q worms, we found that BW-40Q worms (containing a disease-length polyglutamine tract) exhibit mitochondrial fragmentation (Fig. 2A) and have disrupted mitochondrial networks (Supplementary Fig. 4), while BW-24Q worms (containing an unaffected-length polyglutamine tract) have elongated, tubular mitochondria, similar to wild-type worms (Fig. 2A; Supplementary Fig. 4). Like BW-Htt74Q worms, BW-40Q worms have increased mitochondrial number (Fig. 2B) and decreased mitochondrial area (Fig. 2C) compared to wild-type worms. The levels of mtDNA in BW-40Q worms are equivalent to wild-type levels (Supplementary Fig. 3), suggesting that the increase in mitochondrial number results from mitochondrial fragmentation. In addition, mitochondria in BW-40Q worms exhibit increased circularity compared to those in wild-type worms (Fig. 2D). This indicates that the expression of a disease-length polyglutamine tract is sufficient to cause mitochondrial fragmentation in body wall muscle independent of any Htt protein sequence.

**Figure 3.**
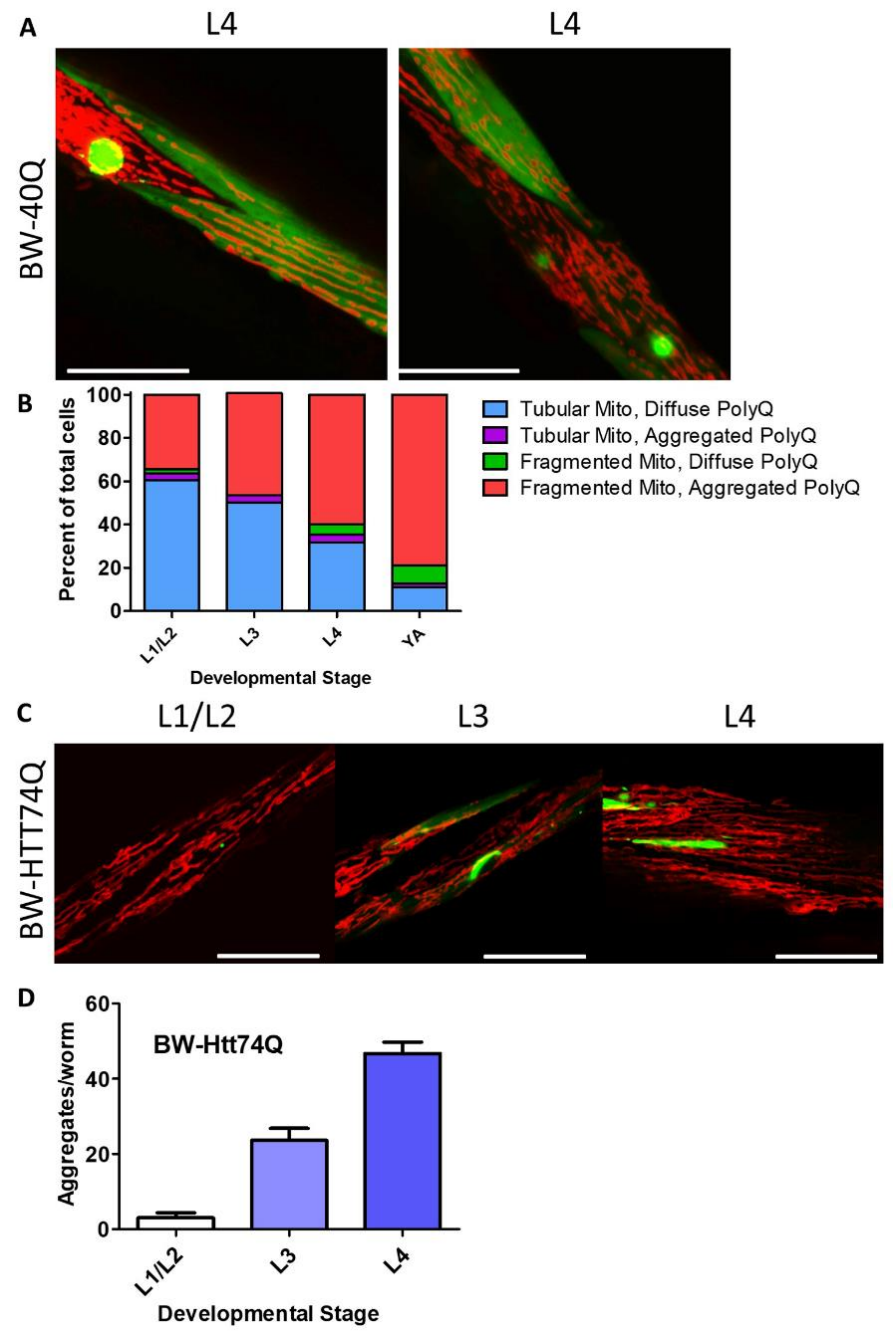
Disruption of mitochondrial network is associated with polyglutamine aggregation. Images show neighboring body wall muscle cells from worms expressing a disease-length polyglutamine tract in body wall muscles (BW-40Q worms) at the L4 stage of development. One cell has diffuse expression of the polyglutamine (PolyQ) protein and normal, tubular mitochondrial networks, while the other cell has a polyglutamine aggregate and a disrupted mitochondrial network (A). Initially, body wall muscle cells have tubular mitochondria and diffuse polyglutamine protein. Over time, an increasing number of body wall muscle cells have fragmented mitochondria and aggregated polyglutamine. Very few cells exhibit mitochondrial fragmentation and diffuse polyglutamine protein expression, or tubular mitochondria with aggregated polyglutamine protein (B). In BW-Htt-74Q worms, mitochondrial morphology is similar to that in wild-type worms during early development (C). Polyglutamine protein aggregation increases throughout development in BW-Htt74Q worms (D). The images in panels A and C are compressed z-stacks collected on a confocal microscope. Scale bars indicate 25 µM. A minimum of three biological replicates were performed. Bars indicate the mean value. Error bars indicate SEM.

### Mitochondrial fragmentation is associated with polyglutamine aggregation

In imaging mitochondrial morphology in worm models of HD, we observed that, during development, neighboring muscle cells could exhibit different mitochondrial morphologies. While some cells exhibited parallel tracts of elongated mitochondria in combination with diffuse expression of polyglutamine protein, adjacent cells had fragmented or disorganized mitochondrial networks and aggregated polyglutamine protein (Fig. 3A). We rarely observed the co-occurrence of disrupted mitochondrial networks and diffuse polyglutamine localization.

To further explore this relationship, we performed a time course examining mitochondrial morphology and polyglutamine protein aggregation throughout development in BW-40Q worms (Fig. 3B). Initially most cells had tubular, elongated mitochondria and diffuse polyglutamine protein. Over time, the number of cells exhibiting this phenotype declined, while an increasing number of cells had fragmented mitochondria and aggregated polyglutamine protein. Throughout the time course we observed few cells with fragmented mitochondria and diffuse polyglutamine protein, or tubular mitochondria and aggregated polyglutamine protein. This suggests that polyglutamine aggregation and mitochondrial fragmentation are temporally-linked events.

To extend these findings to another model, we examined aggregation and mitochondrial morphology throughout development in BW-Htt74Q worms. We found that during early development (developmental stages from L1 to L4), mitochondria in BW-Htt74Q worms are elongated and exist in parallel tracts (Fig. 3C), similar to mitochondria in wild-type worms, but become fragmented by the time worms reached adulthood (see Fig. 1A). At the same time, BW-Htt74Q worms initially have diffuse expression of the Htt exon 1 fragment protein but show increased aggregation with age (Fig. 3D). Thus, as in BW-40Q worms, both aggregation and mitochondrial fragmentation increase with age, and both tend to occur together in the same cell. Combined, this indicates that mitochondrial fragmentation is strongly associated with polyglutamine aggregation.

**Figure 4.**
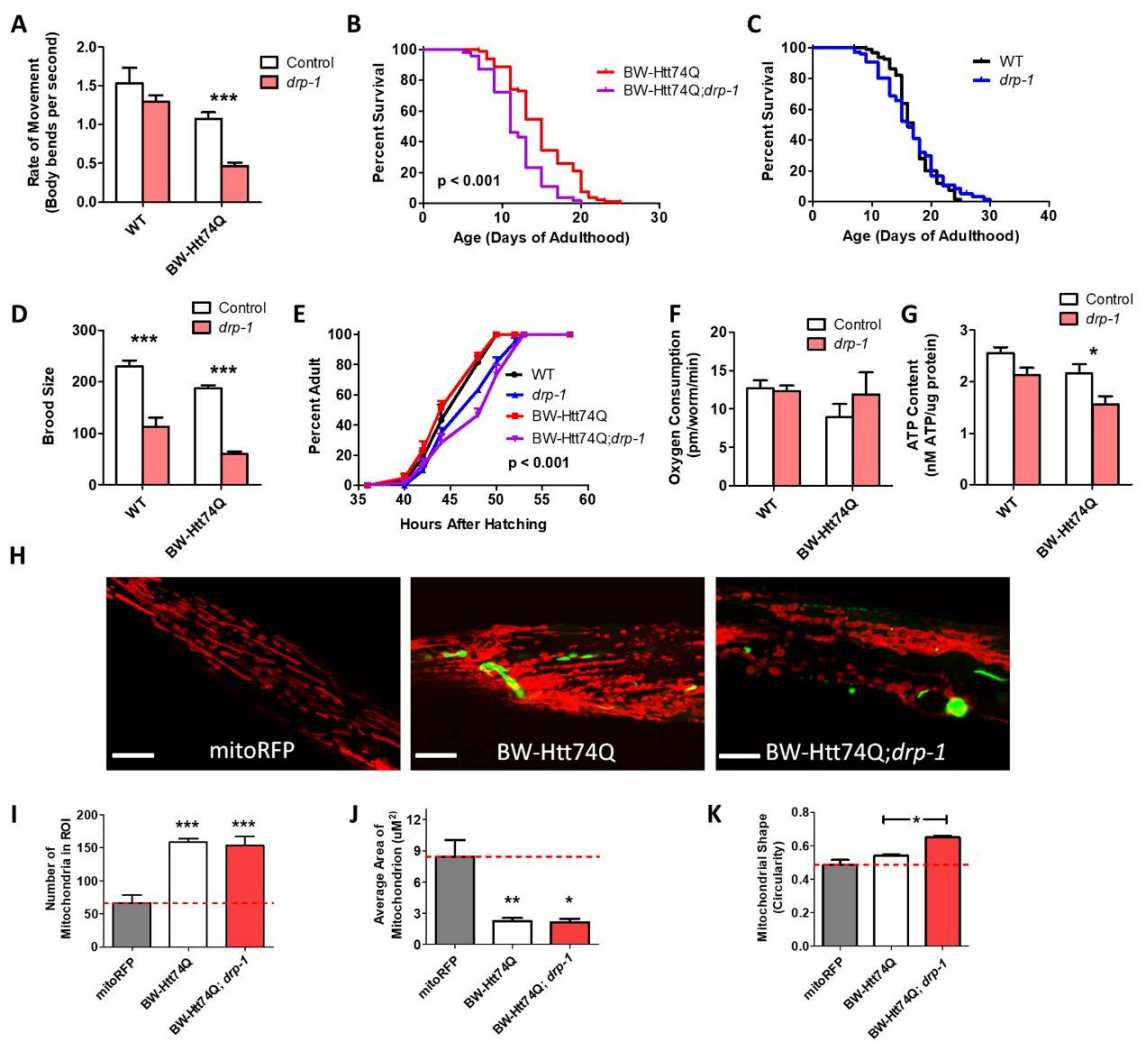
Inhibition of mitochondrial fission has detrimental effects in *C. elegans* models of Huntington’s disease expressing expanded polyglutamine tracts in body wall muscle. To examine the effect of disrupting mitochondrial fission in worm models of HD, a body wall muscle (BW-Htt74Q worms) model of HD was crossed to a *drp-1* deletion mutant. The *drp-1* mutation significantly decreased movement (A) and lifespan (B) in BW-Htt74Q worms but had no effect on wild-type worms (A, C). Loss of *drp-1* resulted in decreased fertility (D) and slower post-embryonic development (E) in both BW-Htt74Q and wild-type worms. While the *drp-1* deletion did not affect oxygen consumption (F) in either genotype, it resulted in a decreased levels of ATP (G). Deletion of *drp-1* did not decrease the mitochondrial fragmentation that is present in BW-Htt74Q worms (H), as indicated by quantification of mitochondrial number (I), mitochondrial area (J) and mitochondrial shape (K). Note that we used wild-type worms as a control instead of BW-Htt28Q worms because we observed the formation of aggregates in BW-Htt28Q worms, which could complicate the interpretation of the results. The images in panel H are compressed z-stacks collected on a confocal microscope. Scale bars indicate 10 µM. A minimum of three biological replicates were performed. Bars indicate the mean value. One-way ANOVA was used to assess significance in H, I and J. Two-way ANOVA was used to assess significance in A, C, E, and F. Log-rank test was used to assess significance in B. Repeated measures ANOVA was used to assess significance in D. Error bars indicate SEM. ROI - region of interest. *p<0.05, **p<0.01, ***p<0.001.

### C. elegans models of Huntington’s disease have increased expression of mitochondrial fission and fusion genes

In order to explore the mechanism underlying the disrupted mitochondrial networks observed in the HD worm models, we used quantitative reverse transcription PCR (qPCR) to measure expression of fission and fusion genes in day 1 adult animals. We found that both HD models in which the disease-length polyglutamine protein is expressed in body wall muscle (BW-Htt74Q, BW-40Q) showed significant changes in the expression of both mitochondrial fission and fusion genes (Supplementary Fig. 5). In both models, *fis-1* and *eat-3* mRNA levels were elevated. It is unclear whether these changes in gene expression contribute to the disruption of mitochondrial networks observed in these strains, or whether these genes are activated in an attempt to restore wild-type mitochondrial morphology.

### Disruption of mitochondrial fission can be detrimental in a body wall muscle model of Huntington’s disease

Having shown that worm models of HD exhibit increased mitochondrial fragmentation, we next sought to determine if decreasing mitochondrial fission would help to restore mitochondria morphology, and whether this would ameliorate phenotypic deficits present in these worms. To decrease mitochondrial fission, we crossed BW-Htt74Q worms to a *drp-1* deletion mutant (*tm1108*) [42]. In worms, *drp-1* is expressed highly in body wall muscle and neurons [43], making it a good genetic target for the worm models expressing an expanded polyglutamine tract in body wall muscle. In these experiments, we used wild-type worms as a control instead of BW-Htt28Q worms because we observed the formation of polyglutamine aggregates in BW-Htt28Q worms. Since this length of polyglutamine tract does not aggregate in most HD models, and is within the unaffected range in humans, the aggregation in BW-Htt28Q worms could complicate the interpretation of the results.

Surprisingly, we found that the *drp-1* deletion resulted in decreased movement, as measured by the rate of thrashing in liquid of day 1 young adult animals (Fig. 4A), and decreased lifespan (Fig. 4B) in BW-Htt74Q worms, but had no significant effect on these phenotypes in wild-type worms (Fig. 4A, C). Although we and others find that *drp-1* mutants have a wild-type lifespan [44, 45], it should be noted that one report has indicated that *drp-1* mutants are short-lived compared to wild-type worms [46]. The *drp-1* deletion also decreased fertility, as measured by self-brood size (Fig. 4D) and slowed development (Fig. 4E) in both BW-Htt74Q worms and wild-type worms. Examining the effect of the *drp-1* deletion on mitochondrial function revealed no effect on the rate of oxidative phosphorylation, as measured by oxygen consumption (Fig. 4F), but caused a small decrease in ATP levels (Fig. 4G). Finally, we found that disruption of *drp-1* did not decrease the mitochondrial fragmentation present in BW-Htt74Q worms (Fig. 4H-K). Taken together, these results suggest that inhibition of mitochondrial fission can be detrimental in a body wall muscle model of HD.

### drp-1 deletion increases expression of polyglutamine transgene

It was previously reported that RNAi against *drp-1* increases expression of the polyglutamine transgene [16]. As increasing the levels of the polyglutamine protein would be expected to increase toxicity, we sought to determine whether *drp-1* deletion also resulted in increased transgene expression. Accordingly, we used qPCR to measure the levels of polyglutamine transgene expression in BW-Htt74Q worms compared to BW-Htt74Q;*drp-1* worms. We found that BW-Htt74Q;*drp-1* worms showed a 92% increase in Htt74Q:GFP mRNA compared to BW-Htt74Q worms (Supplementary Fig. 6). This increase in polyglutamine expression could contribute to the detrimental effects of *drp-1* deletion in this strain.

### Decreasing mitochondrial fission through drp-1 RNAi can be beneficial in a body wall muscle model of Huntington’s disease

In contrast to our results obtained with a *drp-1* deletion, another group previously observed that RNAi against *drp-1* improved movement in BW-Htt74Q worms [16]. Since a complete loss of DRP-1 can lead to a wide range of abnormalities [47], we wondered if the *drp-1* deletion has detrimental effects that are independent of, or that synergize with polyglutamine toxicity, which masked a potential beneficial effect of decreasing *drp-1* levels. Accordingly, we investigated whether decreasing levels of *drp-1* by RNAi would be more beneficial in the HD model. To do this, we fed BW-Htt74Q worms with RNAi bacteria that directly targets *drp-1.* The RNAi was administered using an L4 parental protocol, in which RNAi knockdown is begun at the L4 stage of the parental generation prior to testing their progeny (the experimental generation). Using this paradigm, *drp-1* RNAi-treated worms exhibited *drp-1* mRNA levels of approximately 30% of empty vector (EV) controls, and there was no difference in the knockdown efficiency between wild-type and BW-Htt74Q worms (Supplementary Fig. 7).

**Figure 5.**
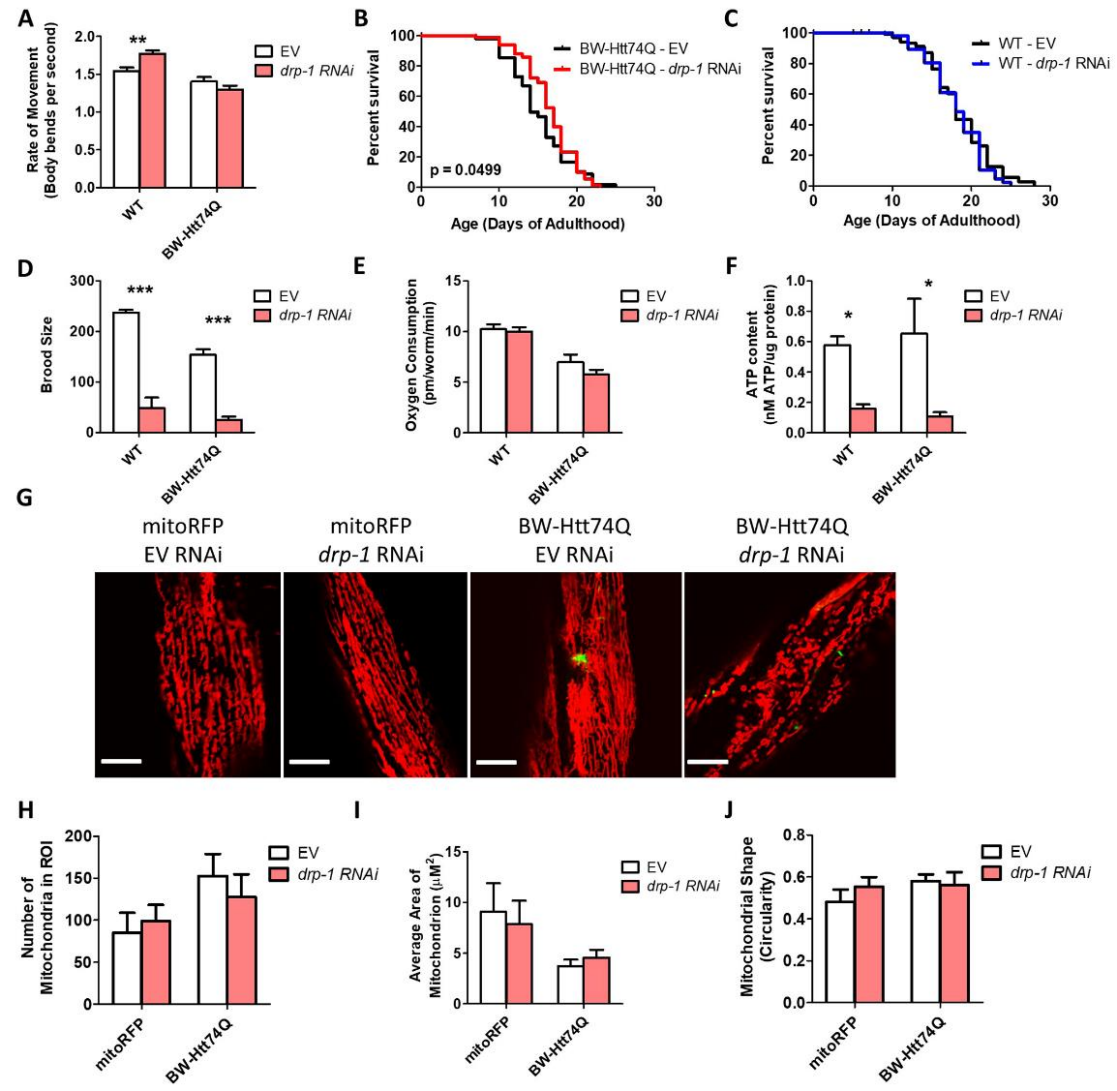
Decreasing expression of mitochondrial fission protein DRP-1 through RNAi increases lifespan in body wall muscle model of Huntington’s disease. The rate of movement in BW-Htt74Q worms is unchanged with *drp-1* RNAi (A). Knocking down *drp-1* results in a small increase in lifespan in BW-Htt74Q worms (B), but has no effect in wild-type worms (C). *drp-1* RNAi decreases fertility in wild-type and BW-Htt74Q worms (D). While *drp-1* RNAi did not affect oxygen consumption in wild-type or BW-Htt74Q worms (E), knockdown of *drp-1* reduced ATP levels in both strains (F). Knocking down *drp-1* expression using RNAi does not affect mitochondrial morphology in control mitoRFP worms and does not restore tubular mitochondrial networks in BW-Htt74Q worms (G). Quantification of mitochondrial morphology reveals no significant changes in mitochondrial number (H), mitochondrial size (I) or mitochondrial shape (J) after treatment with *drp-1* RNAi. The images in panel G are compressed z-stacks collected on a confocal microscope. Scale bars indicate 10 µM. A minimum of three biological replicates were performed. Bars indicate the mean value. Significance was assessed using two-way ANOVA (A,C,D,E,G,H,I) or log-rank test (B,C). Error bars indicate SEM. ROI - region of interest. *p<0.05, **p<0.01,***p<0.001.

Unlike the *drp-1* mutation, *drp-1* RNAi did not decrease the rate of movement in BW-Htt74Q worms (Fig. 5A) and resulted in a small but significant increase in lifespan in these worms (Fig. 5B, C). This suggests that the detrimental effect of the *drp-1* mutation on movement in BW-Htt-74Q worms requires *drp-1* levels to be reduced beyond a specific threshold or be completely absent. Like the *drp-1* mutation, both wild-type and BW-Htt74Q worms treated with *drp-1* RNAi have markedly decreased brood size compared to worms grown on EV RNAi (Fig. 5D).

To determine whether the mild improvement in lifespan was associated with changes in mitochondrial function, we quantified the effect of *drp-1* RNAi on mitochondrial form and function. While there was no effect of *drp-1* RNAi on oxygen consumption in wild-type or BW-Htt74Q worms (Fig. 5E), it did result in decreased ATP levels (Fig. 5F). As with the *drp-1* mutation, the disrupted mitochondrial networks present in BW-Htt74Q worms were not rescued by *drp-1* RNAi (Fig. 5G). Mitochondrial number, area, and circularity were all unchanged in BW-Htt74Q worms treated with *drp-1* RNAi compared to EV (Fig. 5H-J).

**Figure 6.**
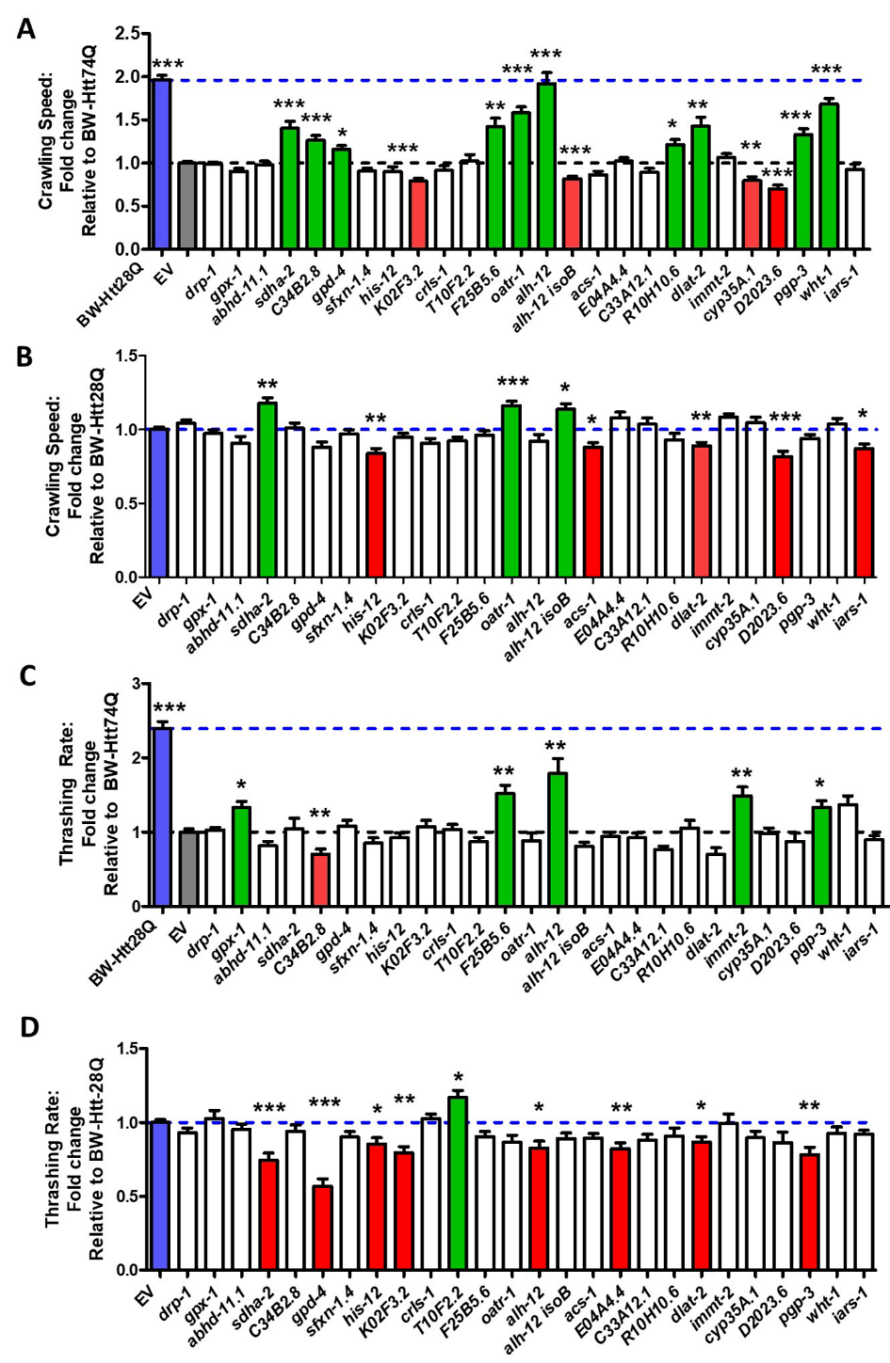
RNAi clones that decrease mitochondrial fragmentation improve movement in body wall muscle model of Huntington’s disease. BW-Htt74Q and BW-Htt28Q control worms were treated with RNAi against genes that were previously shown to decrease mitochondrial fragmentation when knocked down by RNAi. Movement was then assessed by crawling and thrashing assays using an unbiased video-tracking automated system. Ten of the 25 RNAi clones that decrease mitochondrial fragmentation were found to increase the crawling rate in BW-Htt74Q worms (A). Of these 10 RNAi clones, two RNAi clones also increased crawling speed in BW-Htt28Q control worms, indicating that 8 RNAi clones specifically improve movement in the disease model (B). Five of the 25 RNAi clones that decrease mitochondrial fragmentation increased the rate of movement in liquid (thrashing rate) of BW-Htt74Q worms (C). Only one of the RNAi clones increased the thrashing rate of BW-Htt28Q worms (D). Three RNAi clones, *F25B5.6, alh-12* and *pgp-3* increased both crawling speed and thrashing rate in BW-Htt74Q worms. Blue bars show BW-Htt28Q worms treated with empty vector (EV). Grey bars show BW-Htt74Q worms treated with empty vector. Green bars show RNAi clones that significantly increased movement. Red bars show RNAi clones that significantly decreased movement. Blue dotted line shows rate of movement for BW-Htt28Q treated with EV. Grey dotted line shows rate of movement for BW-Htt74Q treated with EV. Bars indicate the mean value. Significance was assessed using one-way ANOVA. Error bars indicate SEM. *p<0.05, **p<0.01, ***p<0.001.

**Table 1:** RNAi clones that improved movement in *C. elegans* models of Huntington’s disease.

Target gene	*Drosophila* homolog	Mammalian homolog	Effect on crawling BW-Htt74Q	Effect of crawling BW-Htt28Q	Effect on thrashing BW-Htt74Q	Effect of thrashing BW-Htt28Q	Effect on aggregation
*drp-1*	*Drp1*	*DNM1L*	No effect	No effect	No effect	No effect	Decreased
*sdha-2*	*SdhA*	*SdhA*	Increased	Increased	No effect	Decreased	No effect
*C34B2.8*	*ND-B16.6*	*NDUFA13*	Increased	No effect	Decreased	No effect	No effect
*gpd-4*	*Gapdh2*	*GAPDH*	Increased	No effect	No effect	Decreased	No effect
*F25B5.6*	*Fpgs*	*FPGS*	Increased	No effect	Increased	No effect	No effect
*oatr-1*	*Oat*	*OAT*	Increased	Increased	No effect	No effect	No effect
*alh-12*	*Aldh*	*ALDH9A1*	Increased	No effect	Increased	Decreased	No effect
*R10H10.6*	*CG2846*	*RFK*	Increased	No effect	No effect	No effect	No effect
*dlat-2*	*muc*	*DLAT*	Increased	Decreased	No effect	Decreased	No effect
*pgp-3*	*Mdr49*	*ABCB4*	Increased	No effect	Increased	Decreased	No effect
*wht-1*	*w*	*ABCG1*	Increased	No effect	No effect	No effect	No effect
*gpx-1*	*PHGPx*	*GPX4*	No effect	No effect	Increased	No effect	No effect
*immt-2*	*Mitofilin*	*IMMT*	No effect	No effect	Increased	No effect	No effect

### Decreasing mitochondrial fragmentation through multiple genetic targets rescues movement deficits in a body wall muscle model of Huntington’s disease

Given that DRP-1 is the main protein required for mitochondrial fission and we observed detrimental effects of disrupting *drp-1* in both wild-type worms and worm models of HD, inhibiting DRP-1 might not be an ideal therapeutic target for HD. Accordingly, we explored the therapeutic potential of other genetic targets that decrease mitochondrial fragmentation. A previous study performed a targeted RNAi screen to examine the effect of knocking down mitochondria-associated genes on mitochondrial morphology [48]. In this study, they examined 719 genes predicted to encode mitochondrial proteins and identified 25 RNAi clones that decrease mitochondrial fragmentation in body wall muscle. We performed a targeted RNAi screen to examine the effect of these 25 RNAi clones in BW-Htt74Q worms. Treatment with RNAi was begun at the L4 stage of the parental generation and the rate of movement was assessed in the progeny (experimental generation). The rate of movement was assessed by unbiased video-tracking of movement on solid plates (crawling) and movement in liquid (thrashing).

**Figure 7.**
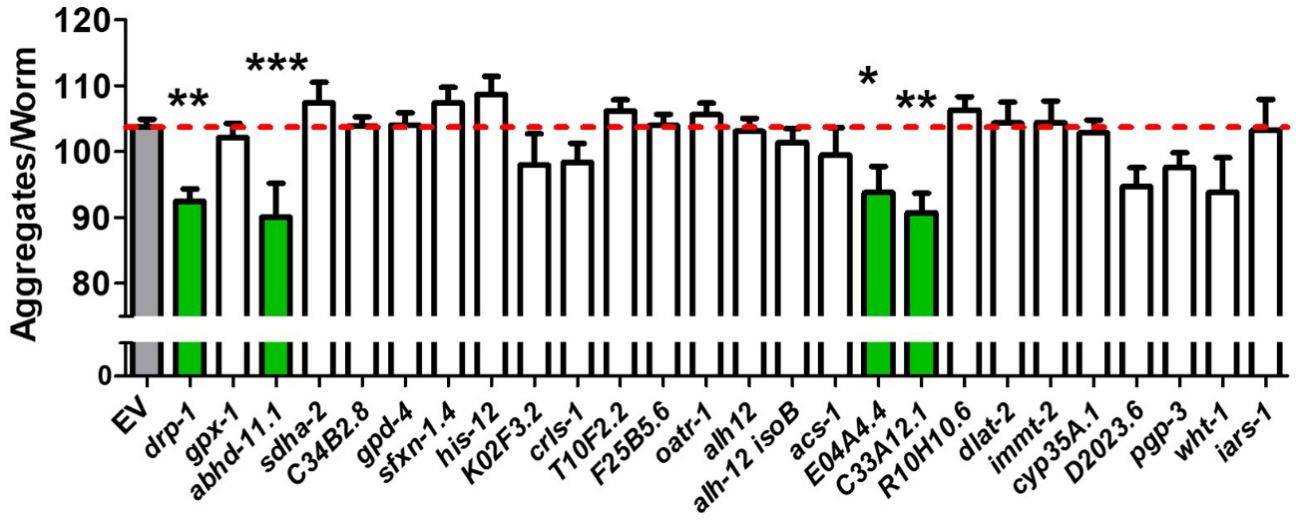
Most RNAi clones that decrease mitochondrial fragmentation do not cause a decrease in polyglutamine aggregation. BW-40Q worms were treated with RNAi against genes which decrease mitochondrial fragmentation in *C. elegans* when knocked down via RNAi. Animals were imaged as day 1 adults. Of the 26 RNAi clones tested, four RNAi clones resulted in a small but significant decrease in total aggregates per worm: *drp-1, abhd-11.1, E04A4.4,* and *C33A12.1.* Grey bars show BW-40Q worms treated with empty vector (EV). Green bars show RNAi clones that significantly decreased aggregate number. None of the RNAi clones resulted in increased aggregation. Bars indicate the mean value. Significance was assessed using one-way ANOVA. Error bars indicate SEM. *p<0.05, **p<0.01, ***p<0.001.

We found that 10 of the 25 RNAi clones that decrease mitochondrial fragmentation increase crawling speed in BW-Htt74Q worms (Fig. 6A). Of these 10 RNAi clones, two clones also increased movement in BW-Htt28Q worms suggesting a non-specific beneficial effect on movement (Fig. 6B). Thus, eight RNAi clones specifically rescued movement deficits in worm models of HD (Table 1). In examining the effect of these same 25 RNAi clones on movement in liquid, we found that five of these clones significantly increased the thrashing rate in BW-Htt74Q worms (Fig. 6C), while a separate clone improved movement in BW-Htt28Q control worms (Fig. 6D). Of the five clones that improved thrashing, three of them, *F25B5.6*, *alh-12* and *pgp-3*, also improved crawling speed (Table 1).

### Decreasing mitochondrial fragmentation does not affect polyglutamine aggregation

To better understand how the RNAi clones that suppress mitochondrial fragmentation caused a decrease in motility defects, we examined the effect of these clones on polyglutamine aggregation. We found that *drp-1* RNAi caused a small but statistically significant decrease in the number of aggregates, as did three of the other RNAi clones (*E04A4.4*, *C33A12.1*, *abhd-11.1*). However, all of these clones were also found to disrupt development and may be suppressing aggregation by slowing development. As none of these RNAi clones rescued motility deficits in the HD worm model, this indicates that decreasing aggregation is not sufficient to improve movement. Importantly, we found that none of the RNAi clones which did suppress motility defects caused a decrease in aggregation levels (Fig. 7, Table 1). Thus, the ability of these clones to rescue movement deficits is not through a decrease in aggregation.

**Figure 8.**
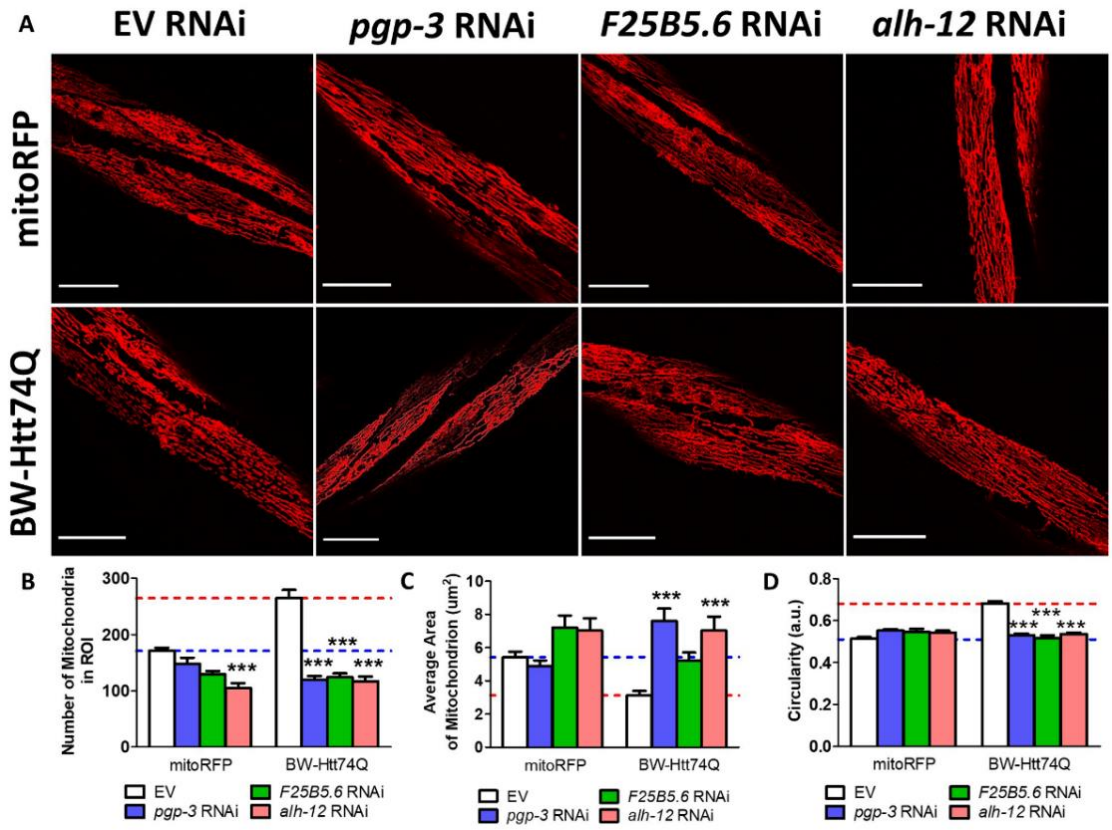
RNAi clones that improve movement correct mitochondrial fragmentation in worm model of Huntington’s disease. BW-Htt74Q worms or mitoRFP control worms were treated with RNAi clones that were shown to improve crawling speed on solid plates and thrashing rate. In every case, the RNAi clones decreased mitochondrial fragmentation such that mitochondrial morphology in the treated worms was equivalent to mitoRFP controls (A). Treatment with RNAi against *pgp-3, F25B5.6* or *alh-12* significantly decreased the number of mitochondria in the region of interest (ROI) (B), significantly increased mitochondrial area (C), and significantly decreased mitochondrial circularity (D). The images shown in panel A are a compressed z-stack collected on a confocal microscope. Scale bar indicates 25 µM. Bar graphs indicate the mean value. Significance shown is the difference from the respective EV RNAi control, and was assessed using one-way ANOVA. Segmentation of mitochondria for quantification was performed using ImageJ’s segmentation and quantification of subcellular shapes (SQUASSH) tool. Average number of mitochondria, average mitochondrial area and average mitochondrial circularity were then measured using the analyze particles tool on ImageJ. Error bars indicate SEM. ***p<0.001.

### Amelioration of movement deficits is associated with restoration of wild-type mitochondrial morphology

To further characterize the three RNAi clones that rescued the movement deficits in BW-Htt74Q worms, we examined the effect of these genes on mitochondrial morphology and lifespan. We found that the mitochondria of BW-Htt74Q worms treated with *pgp-3, F25B5.6* or *alh-12* RNAi was indistinguishable from the mitochondria from mitoRFP control worms (Fig. 8A). The RNAi-treated BW-Htt74Q worms exhibited elongated, tubular mitochondria with no signs of mitochondrial fragmentation. In quantifying the effect of these three RNAi clones on mitochondrial morphology in BW-Htt74Q worms, we found that *pgp-3, F25B5.6* or *alh-12* RNAi significantly decreased mitochondrial number (Fig. 8B), increased average mitochondrial area (Fig. 8C), and decreased mitochondrial circularity (Fig. 8D).

Finally, we examined the effect of *pgp-3, F25B5.6* or *alh-12* RNAi on the lifespan of BW-Htt74Q worms. We found that treatment with these three RNAi clones was unable to restore BW-Htt74Q lifespan to wild-type length (Supplementary Fig. 8). Combined, this suggests that decreasing mitochondrial fragmentation in BW-Htt74Q worms through treatment with RNAi against *pgp-3, F25B5.6* or *alh-12* increases healthspan, as measured by movement in liquid and on solid plates, but not overall lifespan.

## DISCUSSION

### Expression of a disease-length polyglutamine tract in C. elegans causes mitochondrial fragmentation

While the genetic cause of HD was identified in 1993, there are still no treatments available for patients with this devastating disorder that can alter disease progression [49]. Based on several observations linking HD and mitochondrial dysfunction, correcting mitochondrial deficits has been the focus of much research on disease-modifying therapies for HD [50]. Accumulating evidence indicates that mitochondrial dynamics are disrupted in HD [16-23]. We have extended these findings to show that mitochondrial networks are also disrupted in two different *C. elegans* models of HD in which a disease-length polyglutamine tract is expressed in body wall muscle. In both BW-Htt74Q and BW-40Q worms we observed mitochondrial fragmentation and mitochondrial network disorganization leading to an increased number of smaller, more rounded mitochondria, with no increase in mtDNA content. The increase in mitochondrial fragmentation may be a precursor to mitophagy in order to replace mitochondria that are damaged by polyglutamine toxicity or could be a direct effect of the expanded polyglutamine protein. The fact that we observe increased expression of mitochondrial fission genes suggests that the increase in mitochondrial fragmentation might be at least partially due to an active process to increase mitochondrial fission.

Importantly, our results indicate that polyglutamine toxicity can cause mitochondrial fragmentation independently of the huntingtin protein, as a worm model expressing a pure polyglutamine tract linked to YFP also exhibited disrupted mitochondrial networks (BW-40Q worms). Consistent with this idea, mitochondrial fragmentation has been observed in models of other polyglutamine toxicity disorders including Spinocerebellar ataxia 3 (SCA3), Spinocerebellar ataxia 7 (SCA7) and Spinal and bulbar muscular atrophy (SBMA) [51-53]. Combined, this suggests that expression of an expanded polyglutamine tract (or the presence of a CAG repeat expansion in the DNA/RNA) may be sufficient to cause mitochondrial fragmentation independently of the surrounding protein context.

### DRP1 may not be an ideal therapeutic target for Huntington’s disease

Based on the observation of mitochondrial fragmentation in HD patients and models, multiple groups have explored the effect of decreasing the levels or activity of DRP-1 in various models of HD [16, 17, 19, 21]. In each case, decreasing the levels or activity of DRP-1 showed a beneficial effect in HD models. However, in thinking about developing a treatment for HD, DRP1 may not be an ideal target. DRP1 is the main GTPase responsible for mitochondrial fission, which is crucial for proper cellular function. Consistent with this, a number of studies have indicated that loss of DRP1 function can be detrimental [46, 47, 54-56]. For example, loss-of-function mutations in the gene that encodes DRP1 causes a wide range of abnormalities in humans, including epilepsy and encephalopathy [57]. Our results indicate that deletion of *drp-1* can be detrimental in wild-type worms (slow development, decreased fertility) and exacerbate phenotypic deficits in a body wall muscle model of HD (decrease movement, shorten lifespan). Because of the potential negative side effects of directly targeting DRP1, it may be important to explore other approaches to decrease mitochondrial fragmentation as potential therapeutic strategies for HD.

Alternatively, it may be necessary to precisely control the level of *drp-1* disruption. While deletion of *drp-1* had detrimental effects in BW-Htt74Q worms, we found that decreasing *drp-1* levels through RNAi resulted in a small but beneficial effect on lifespan. Similarly, a previous study found that *drp-1* RNAi can increase movement in the same BW-Htt74Q worms [16]. This suggests that the precise level of *drp-1* depletion may need to be controlled to observe a beneficial effect. The difference between *drp-1* deletion and *drp-1* RNAi could also have resulted from the fact that neurons in *C. elegans* have decreased sensitivity to RNAi. In addition to identifying the optimal level of *drp-1* knockdown, it may be possible to minimize detrimental side effects of disrupting *drp-1* by knocking it down in specific tissues, which could be tested using tissue-specific RNAi strains, or by knocking it down during specific periods of development or adulthood, which could be done by administering the RNAi bacteria targeting *drp-1* during specific periods of time.

### Novel therapeutic targets for Huntington’s disease aimed at reducing mitochondrial fragmentation

Because of the potential detrimental effects of targeting DRP-1, we examined 25 other genes which also suppress mitochondrial fragmentation when knocked down using RNAi. Eight of the RNAi clones improved crawling only in the Htt-74Q strain, while five clones improved thrashing only in the Htt-74Q strain. Combined, we identified three RNAi clones (*pgp-3*, *F25B5.6*, and *alh-12*) that improved motility in both assays. These three RNAi clones were also able to completely restore mitochondria morphology in BW-Htt74Q worms to wild-type morphology. These genes represent novel therapeutic targets for HD, which will be important to validate in other models of HD.

The main trait shared between *pgp-3*, *F25B5.6* and *alh-12* are that they cause elongated mitochondria when knocked down by RNAi [48]. *pgp-3* is a p-glycoprotein and performs ATP-dependent export of toxins and xenobiotics out of the cytoplasm. It is required for drug resistance to colchicine and chloroquine and upregulated when animals are exposed to heavy metals [58, 59]. *pgp-3* is conserved and has a human orthologue, ABCB4 (ATP binding cassette subfamily B member 4), which is upregulated in an R6/2 mouse model of HD [60].

F25B5.6 also has ATP-binding activity and is predicted to be a folylpolyglutamate synthase. The human homologue, FPGS, is a mitochondrial enzyme, which maintains folylpolyglutamate concentrations in the cytoplasm and mitochondria. It has not previously been associated with HD or neurodegeneration.

*alh-12* is an aldehyde dehydrogenase, which is upregulated in long-lived *C. elegans* mutants [60]. The human homologue, ALDH9A1, has not been experimentally linked with HD, however a meta-analysis of pathways affected in HD predicted that ALDH9A1 is important for HD as it is involved in multiple metabolic pathways that are affected in HD [61].

As all three of these genes have homologs in mice and humans, a key next step will be to examine the effect of these genes on mitochondrial morphology and polyglutamine toxicity in mammalian models. These validation steps could be performed in mammalian cells or mouse models, which we and others have shown to recapitulate many features of the human disease [62-65].

### Polyglutamine aggregation is associated with mitochondrial fragmentation, but can be experimentally dissociated

While polyglutamine aggregation is associated with toxicity, whether or not aggregation causes toxicity, reduces toxicity or is an epiphenomenon is still debated. Both polyglutamine aggregation and toxicity increase with both age and the length of the glutamine repeat [41]; however, decreases in polyglutamine aggregation do not always cause a decrease in polyglutamine toxicity [66], and the formation of aggregates has been associated with a decreased probability of death [67]. In our study, we show that polyglutamine aggregation is associated with mitochondrial fragmentation. In both the BW-Htt74Q model and BW-40Q model, mitochondria are tubular at hatching and polyglutamine proteins exhibit diffuse localization. By the L4 stage of development, mitochondria start to become fragmented, and this event is temporally correlated with the formation of aggregates. Once worms have reached young adulthood, essentially all muscle cells have fragmented mitochondria and aggregated polyglutamine protein.

Despite the tight correlation between aggregation and mitochondrial fragmentation, our data show that these phenotypes can be experimentally dissociated. Knocking down the expression of *pgp-3, F25B5.6* or *alh-12* prevented mitochondrial fragmentation in BW-Htt74Q worms (Fig. 8) but had no effect on aggregation. This indicates that mitochondrial fragmentation is not required for aggregation.

Similarly, while BW-Htt74Q worms and BW-40Q worms have both movement deficits and polyglutamine aggregation, our data indicates that these phenotypes can be separated. None of the RNAi clones which improved either crawling or thrashing defects in BW-Htt74Q worms caused a decrease in the number of polyglutamine aggregates, while RNAi clones that did decrease aggregation in these worms did not have a beneficial effect on movement. Combined, our results suggest that polyglutamine aggregation is not responsible for the movement deficits in this worm model of HD.

### Conclusions

In this work, we show that *C. elegans* models of HD exhibit mitochondrial fragmentation and disorganized mitochondrial networks, which are associated with polyglutamine aggregation. Our observation that decreasing DRP-1 levels can have detrimental effects in a body wall muscle model of HD, suggests that DRP1 may not be an ideal therapeutic target for HD, or that great care must be taken to ensure that DRP1 levels are only decreased by a certain amount. As an alternative to targeting DRP1, we identified three novel genetic targets (*pgp-3, F25B5.6,* and *alh-12*) that improved both crawling and swimming in a *C. elegans* model of HD. These genetic targets also corrected deficits in mitochondrial morphology, thereby demonstrating that mitochondrial fragmentation can be prevented without disrupting the mitochondrial fission machinery. These results suggest that strategies aimed at correcting mitochondrial fragmentation may be beneficial in the treatment of HD.

## Supplementary Materials

The Supplemenantry data can be found online at: www.aginganddisease.org/EN/10.14336/AD.2021.0404.


