# Supplementary material for: Targeting Mitochondrial Network Disorganization is Protective in C. elegans Models of Huntington’s Disease
Source: Aging Dis. 2021 Oct 1;12(7):1753–72. doi: 10.14336/AD.2021.0404 (PMC8460302; doi:10.14336/AD.2021.0404)
Supplement: Supplementary file 1 [file AD-12-7-1753-s.pdf]

SUPPLEMENTARY DATA

Targeting Mitochondrial Network Disorganization is Protective in *C. elegans* Models of Huntington's Disease

Emily Machiela[1,#], Paige D. Rudich[2,3,#], Annika Traa[2,3,#], Ulrich Anglas[2,3], Sonja K. Soo[2,3], Megan M. Senchuk[1], Jeremy M. Van Raamsdonk[1,2,3,4,5,*]

© 2021. Machiela E et al. Published online at http://www.aginganddisease.org/EN/10.14336/AD.2021.0404

SUPPLEMENTARY DATA

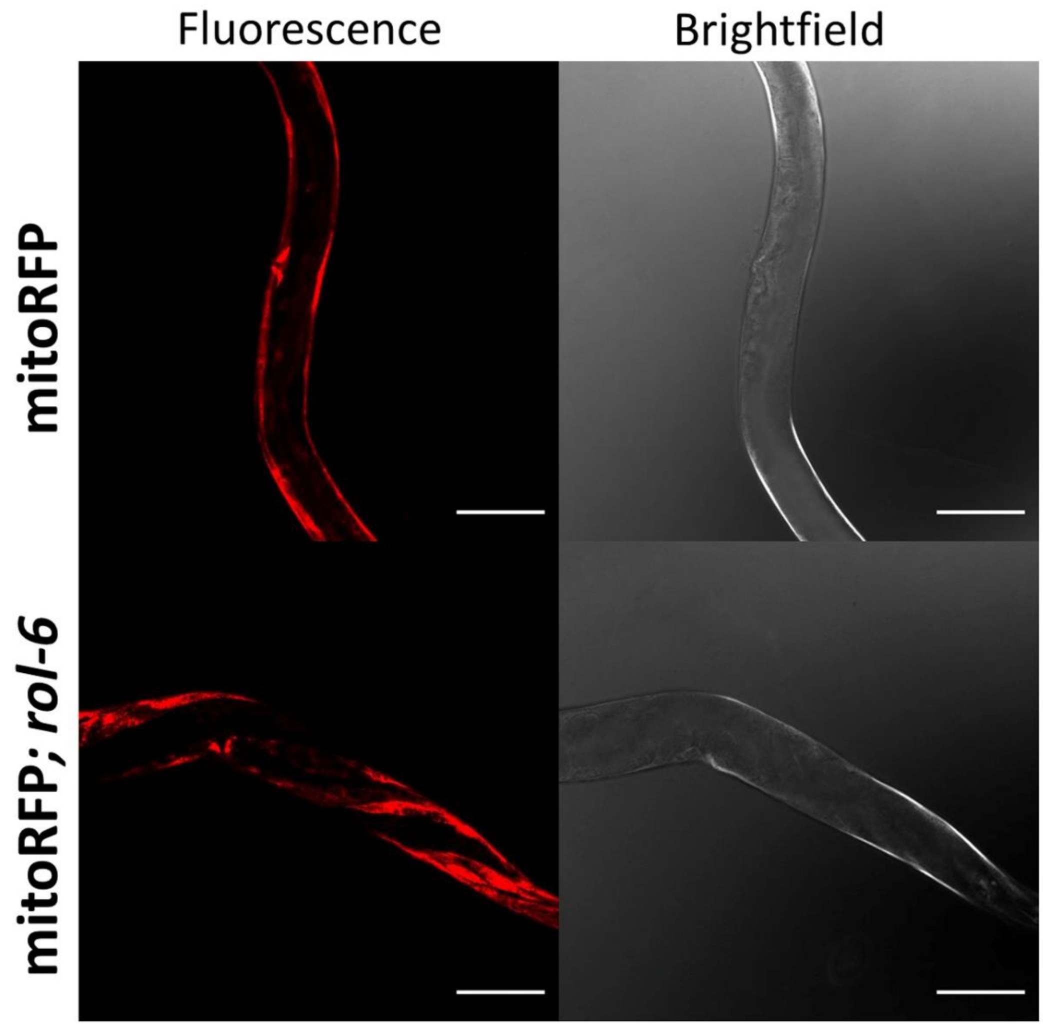

Supplementary Figure 1. The *rol-6* mutation facilitates imaging of body wall muscles in *C. elegans*. The body wall muscles of *C. elegans* are arranged into two segments than run along the length of the worm on either side of the body. When mounted for imaging this results in muscles not facing the microscope lens in many worms (A), therefore making it difficult to image their mitochondria. Introducing the *rol-6* mutation (B), causes worms to move in a twisting motion. When mounted, this results in muscles appearing twisted around the body, with much of the muscle facing the microscope lens and therefore allowing for imaging of the mitochondria. Scale bars represent 25µm.

© 2021. Machiela E et al. Published online at http://www.aginganddisease.org/EN/10.14336/AD.2021.0404

SUPPLEMENTARY DATA

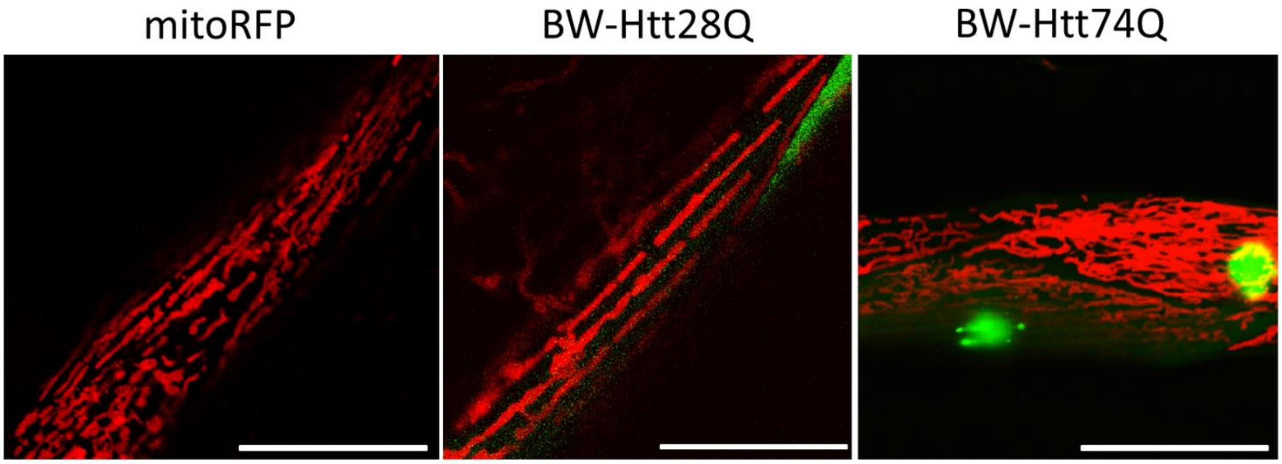

Supplementary Figure 2. BW-Htt74Q worm model of Huntington's disease has disorganized mitochondrial networks. In addition to showing mitochondrial fragmentation (see Figure 1), BW-Htt74Q worms also exhibit disorganized mitochondrial networks. These disorganized networks are rarely observed in BW-Htt28Q or mitoRFP control worms, which have elongated, tubular mitochondria. Mitochondria are labelled with RFP (red), while Htt is labelled with GFP (green). mitoRFP strain is *syIs243[Pmyo-3::TOM20:RFP]*. BW-Htt28Q and BW-Htt74Q worms also express *syIs243[Pmyo-3::TOM20:RFP]* transgene. Scale bars represent 25 µm. These images are compressed z-stacks collected on a confocal microscope.

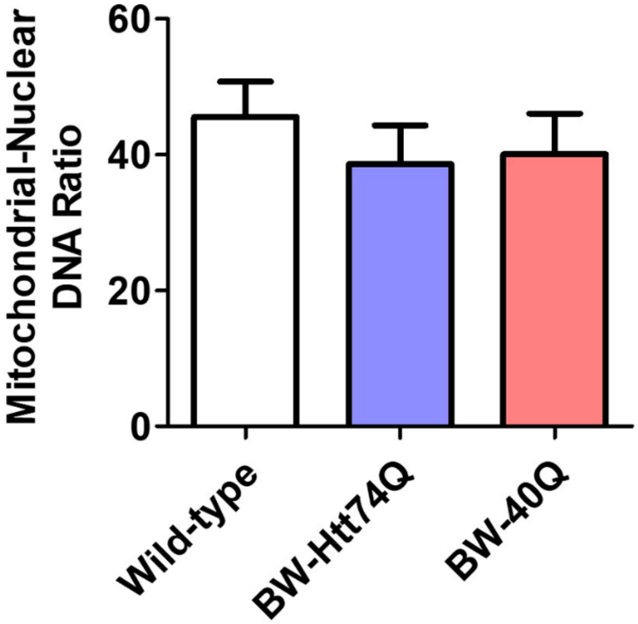

Supplementary Figure 3. BW-Htt74Q worm model of Huntington's disease has disorganized mitochondrial networks. In addition to showing mitochondrial fragmentation (see **Figure 1**), BW-Htt74Q worms also exhibit disorganized mitochondrial networks. These disorganized networks are rarely observed in BW-Htt28Q or mitoRFP control worms, which have elongated, tubular mitochondria. Mitochondria are labelled with RFP (red), while Htt is labelled with GFP (green). mitoRFP strain is *syIs243[Pmyo-3::TOM20:RFP]*. BW-Htt28Q and BW-Htt74Q worms also express *syIs243[Pmyo-3::TOM20:RFP]* transgene. Scale bars represent 25 µm. These images are compressed z-stacks collected on a confocal microscope.

© 2021. Machiela E et al. Published online at http://www.aginganddisease.org/EN/10.14336/AD.2021.0404

SUPPLEMENTARY DATA

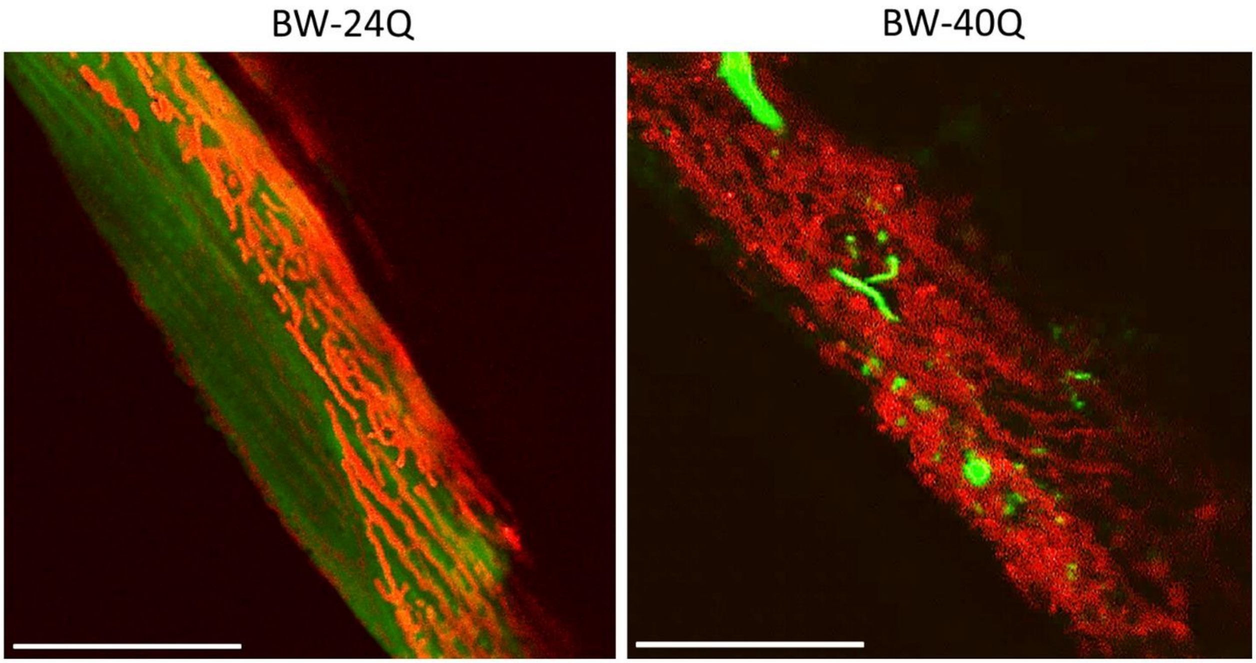

Supplementary Figure 4. BW-40Q worm model of Huntington's disease has disorganized mitochondrial networks. In addition to showing mitochondrial fragmentation (see Figure 2), BW-40Q worms also exhibit disorganized mitochondrial networks. These disorganized networks are rarely observed in BW-24Q control worms, which have elongated, tubular mitochondria. Mitochondria are labelled with RFP (red), while polyglutamine protein is labelled with YFP (green). BW-24Q and BW-40Q worms express *syIs243[Pmyo-3::TOM20:RFP]* transgene. These images are compressed z-stacks collected on a confocal microscope. Scale bars indicate 25 µM.

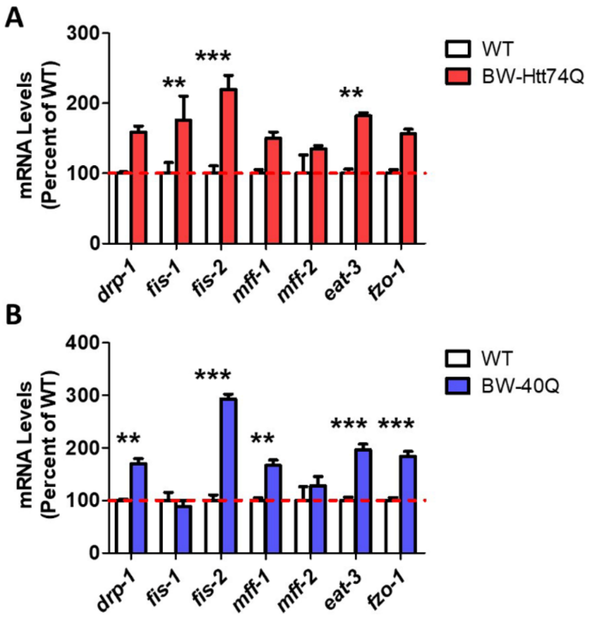

Supplementary Figure 5. Mitochondrial fission and fusion genes are upregulated in body wall muscle models of Huntington's disease. The expression of mitochondrial fission genes (*drp-1, fis-1, fis-2, mff-1, mff-2*) and mitochondrial fusion genes (*eat-3, fzo-1*) was measured using quantitative real time RT-PCR. A. Expression of huntingtin exon 1 with an expanded polyglutamine tract in body wall muscle (BW-Htt74Q) caused increased expression of both mitochondrial fission and fusion genes. B. Similarly, expression of a pure polyglutamine tract with a disease length 40 glutamines in body wall muscle (BW-40Q) also resulted in increased expression of both fission and fusion genes. Bars indicate the mean value of three biological replicates. Two-way ANOVA was used to assess significance. Error bars indicate SEM. **p<0.01, ***p<0.001.

© 2021. Machiela E et al. Published online at http://www.aginganddisease.org/EN/10.14336/AD.2021.0404

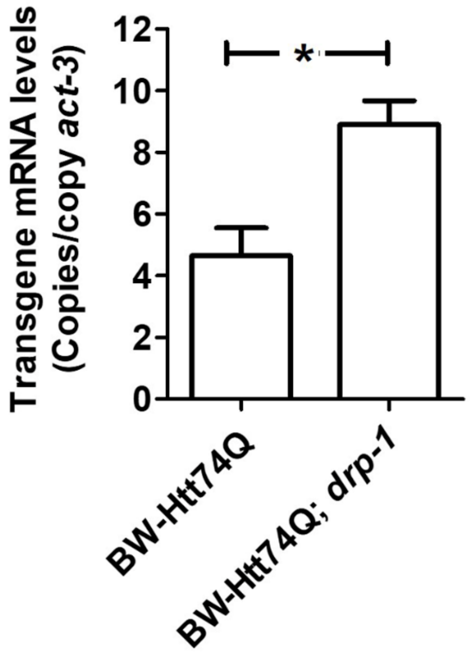

Supplementary Figure 6. Deletion of *drp-1* results in increased expression of the polyglutamine transgene. mRNA was isolated from day 1 pre-fertile young adult worms. Primers were designed to target GFP. Deletion of *drp-1* significantly increased mRNA expression levels of the polyglutamine transgene. Bars indicate mean value of three biological replicates. Statistical significance was assessed using a t-test. Error bars indicate SEM. *p<0.05.

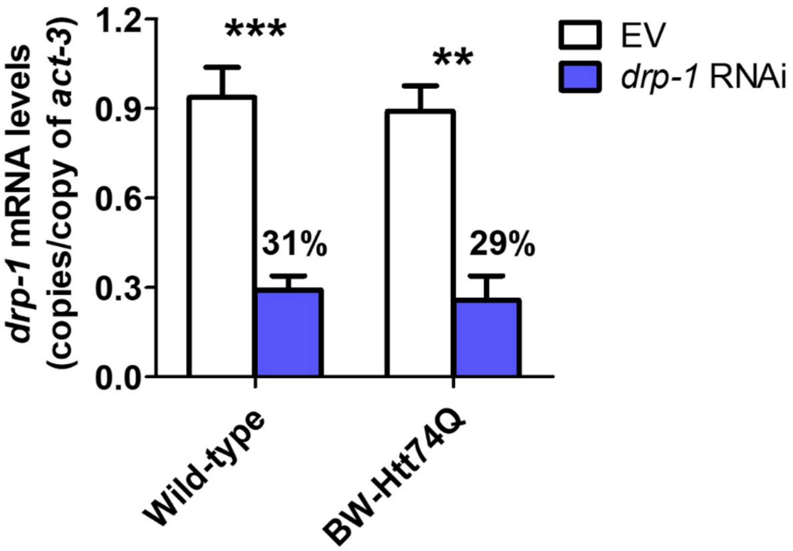

Supplementary Figure 7. RNA interference decreases the levels of *drp-1* mRNA. Wild-type and BW-Htt74Q worms were treated with RNAi bacteria targeting the *drp-1* gene beginning at the L4 stage of the parental generation. *drp-1* levels were measured when the progeny reached the young adult stage using quantitative RT-PCR. In both wild-type and BW-Htt74Q worms, there was a significant decrease in *drp-1* mRNA levels. The level of knockdown was equivalent between the two strains. Bars indicate mean value of three biological replicates. Statistical significance was assessed using a two-way ANOVA with Bonferroni post-hoc test. Error bars indicate SEM. **p<0.01, ***p<0.001.

© 2021. Machiela E et al. Published online at http://www.aginganddisease.org/EN/10.14336/AD.2021.0404

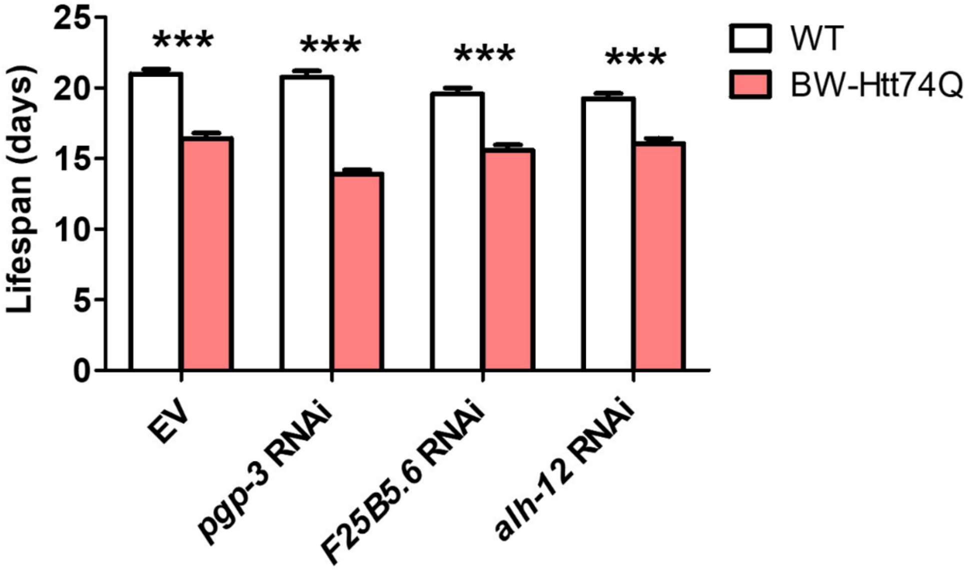

Supplementary Figure 8. RNAi clones that improve movement did not increase lifespan in worm model of Huntington's disease. BW-Htt74Q worms or wild-type (WT) worms were treated with RNAi clones that were shown to improve crawling speed on solid plates and thrashing rate (*pgp-3,* F25B5.6 or *alh-12*). RNAi treatment was begun at the L4 stage of the parental generation and the lifespan of the progeny was measured. None of the three RNAi clones increased the lifespan of BW-Htt74Q worms back to wild-type. Bars indicate mean lifespan. Three biological replicates were performed. Statistical significance was assessed using a two-way ANOVA with Bonferroni post-test. Error bars indicate SEM. ***$p<0.001$.

© 2021. Machiela E et al. Published online at http://www.aginganddisease.org/EN/10.14336/AD.2021.0404
